# Non-Kochen–Specker Contextuality

**DOI:** 10.3390/e25081117

**Published:** 2023-07-26

**Authors:** Mladen Pavičić

**Affiliations:** 1 Center of Excellence for Advanced Materials and Sensors, Research Unit Photonics and Quantum Optics, Institute Ruđer Bošković, 10000 Zagreb, Croatia; mpavicic@irb.hr; 2 Institute of Physics, 10000 Zagreb, Croatia

**Keywords:** quantum contextuality, hypergraph contextuality, MMP hypergraphs, operator contextuality, qutrits, Yu-Oh contextuality, random generation

## Abstract

Quantum contextuality supports quantum computation and communication. One of its main vehicles is hypergraphs. The most elaborated are the Kochen–Specker ones, but there is also another class of contextual sets that are not of this kind. Their representation has been mostly operator-based and limited to special constructs in three- to six-dim spaces, a notable example of which is the Yu-Oh set. Previously, we showed that hypergraphs underlie all of them, and in this paper, we give general methods—whose complexity does not scale up with the dimension—for generating such non-Kochen–Specker hypergraphs in any dimension and give examples in up to 16-dim spaces. Our automated generation is probabilistic and random, but the statistics of accumulated data enable one to filter out sets with the required size and structure.

## 1. Introduction

Quantum contextuality, which precludes assignments of predetermined values to dense sets of states, has found applications in quantum communication [1,2,3], quantum computation [4,5], quantum nonlocality [6], quantum steering [7], and lattice theory [8,9]. Small contextual set experiments were carried out with photons [10,11,12,13,14,15,16,17,18,19,20,21], classical light [22,23,24,25], neutrons [26,27,28], trapped ions [29], solid state molecular nuclear spins [30], and superconducting quantum systems [31].

There are three classes of contextual sets elaborated on in the literature which are not of the more common kind of Kochen–Specker (KS) sets [32,33,34] and for which we provide a hypergraph generalization in this paper.

The first class consists of the operator-based state-independent contextual (SIC) sets put forward by Klyachko et al. [35], Yu and Oh [36], Bengtsson, Blanchfield, and Cabello [37], Xu, Chen, and Su [38], Ramanathan and Horodecki [39], and Cabello, Kleinmann, and Budroni [40], which are not Kochen–Specker sets.

The second class consists of hypergraphs built by multiples of mutually orthogonal vectors where at least one of the multiples contains less than *n* vectors, where *n* is the dimension of space in which a hypergraph resides [4,34,41].

The third class consists of the so-called true-implies-false and true-implies-true sets [42,43].

All sets from these three classes as well as their hypergraph generalization that we elaborate on are contextual, and therefore, we call them non-KS contextual sets.

We provide a general method for arbitrarily generating many non-KS hypergraphs in spaces of up to 16-dim. In order to achieve these goals, we make use of non-binary non-KS McKay–Megill–Pavičić hypergraphs (MMPHs) and their language. By means of our algorithms and programs, we arbitrarily obtain many MMPHs, which can be used for various applications, e.g., to generate new entropic tests of contextuality or new operator-based contextual sets.

The paper is organized as follows.

In Section 2.1, we present the hypergraph language and formalism and define non-binary MMPHs (NBMMPH) and binary MMPHs (BMMPH). We explain how vertices and hyperedges in an MMPH and in *n*-dim space correspond to vectors and their orthogonalities, i.e., *m*-tuples ( 2≤m≤n ) of mutually orthogonal vectors, respectively.

In Section 2.2, we present three methods of generating non-KS MMPHs.

In Section 2.3, we give examples of the aforementioned non-KS sets.

In Section 2.3 and Section 2.4, we generate four- to eight-dim critical non-KS NBMMPHs from master sets, themselves generated from simple vector components.

In Section 2.5 and Section 2.6, we obtain nine- to sixteen-dim critical non-KS NBMMPHs via the dimensional upscaling method, which does not scale up with dimension.

In Section 3, we discuss and review the steps and details of our methods.

In Section 4, we give the technical methods used in the paper.

In Section 5, we summarize the results achieved in the paper.

## 2. Results

We consider a set of quantum states represented by vectors in *n*-dim Hilbert space $H^{n}$ grouped into *m*-tuples ( m≤n ) of mutually orthogonal vectors with m<n holding for at least one *m*. We describe such a set by means of MMPHs. In it, vectors themselves are represented by vertices and mutually orthogonal *m*-tuples of them by hyperedges. However, an MMPH itself has a definition that is independent of a possible representation of vertices by means of vectors. For instance, there are MMPHs without coordinatization, i.e., MMPHs for whose vertices, vectors do not exist. When coordinatization exists, that does not mean that n−m vertices in considered hyperedges do not or cannot exist, but only that we do not take the remaining n−m vertices/vectors into account while elaborating on properties of vertices and hyperedges.

### 2.1. Formalism

Let us define the MMPH formalism/language [34].

**Definition** **1.** *An* MMPH *is an n-dim*

*hypergraph k-l with k vertices and l hyperedges in which*

*1.*  *Every vertex belongs to at least one hyperedge;*
*2.*  *Every hyperedge contains at least two and at most n vertices;*
*3.*  *No hyperedge shares only one vertex with another hyperedge;*
*4.*  *Hyperedges may intersect each other in at most n−2 vertices*
*5.*  *Graphically, vertices are represented as dots, and hyperedges are (curved) lines passing through them.*

**Definition** **2.** *An n-dim* non-binary MMPH (NBMMPH)*, n≥3 ,* [44] *is an* MMPH *for which each hyperedge contains m vertices, 2≤m≤n , and to which it is impossible to assign *1* s and *0* s in such a way that*

*1.*  *No two vertices within any of its edges are both assigned a value of* 1;
*2.*  *In any of its edges, not all of the vertices are assigned a value of* 0.

**Definition** **3.** *An* NBMMPH *in which m=n holds for all hyperedges is a* KS MMPH.

For m = n , an NBMMPH reduces to a KS contextuality set, i.e., to a set satisfying the Kochen–Specker theorem [32,34,45,46].

**Definition** **4.** *An* NBMMPH *in which m<n holds for at least one hyperedge is a* non-KS MMPH.

In this paper, we consider only those non-KS MMPHs for which m=n for at least one hyperedge.

**Definition** **5.** *An n-dim* binary MMPH (BMMPH)*, n≥3 , is an* MMPH *for which each hyperedge contains m vertices, 2≤m≤n , and to which it is possible to assign 1 s and 0 s in such a way that*

*1.*  *No two vertices within any of its edges are both assigned a value of 1;*
*2.*  *In any of its edges, not all of the vertices are assigned a value of 0.*

**Definition** **6.** *A* non-KS NBMMPH *to which vertices are added so as to make the number of vertices equal to n in every hyperedge is called a* filled MMPH.

Filled MMPHs are mostly BMMPHs.

**Definition** **7.** *A* critical NBMMPH *is an* NBMMPH *that is minimal in the sense that removing any of its hyperedges turns it into a* BMMPH.

**Definition** **8.** *Vertex multiplicity is the number of hyperedge vertexes “i” belongs to; we denote it by m(i) .*

**Definition** **9.** *A* master *is a non-critical* MMPH *that contains smaller critical and non-critical sub*-MMPH*s. A collection of sub*-MMPH*s of an* MMPH *master forms its* class.

A *parity proof* ofthe contextuality of a *k*-*l* NBMMPH with odd *l* and where each vertex shares an even number of edges stems from its inherent contradiction: because each vertex shares an even number of hyperedges, there should be an even number of hyperedges with 1 s. At the same time, each edge can contain only one 1 by definition, and since there are an odd number of hyperedges in the MMPH, there should also be an odd number of edges with 1 s.

**Definition** **10.** *A* coordinatization *of a non*-KS NBMMPH *is a set of vectors assigned to its vertices that is a subset of n-dim vectors in $H^{n}$ , n≥3 , assigned to vertices of its filled* MMPH *or its smallest master (they need not coincide) or any of its masters.*

In other words, a “coordinatization” of each hyperedge of a filled MMPH or a smallest master MMPH is represented by an *n*-tuple of orthogonal vectors, while a “coordinatization” of each hyperedge of the original non-KS NBMMPH is represented by a vector *m*-tuple ( m≤n ), which is a subset of that *n*-tuple. This means that the former MMPH inherits its coordinatization from the coordinatization of its master or its filled set (they may, but usually do not, coincide) or any its masters. In our present approach, a coordinatization is automatically assigned to each hypergraph by the very procedure of its generation from master MMPHs, as we show below.

An MMPH is encoded with the help of printable ASCII characters, with the exception of “space”, “0”, “+”, “,” and “.”, organized in single strings; its hyperedges are separated by commas, and each string ends with a period. When all ASCII characters are exhausted, one reuses them prefixed by “+”, and then again by “++”, and so forth. An MMPH with *k* vertices and *l* edges is denoted as a *k*-*l* MMPH. ASCII string representation is used for computer processing. MMPH strings are handled by means of algorithms embedded in the programs SHORTD, MMPSTRIP, MMPSUBGRAPH, VECFIND, STATES01, and others [8,47,48,49,50,51].

### 2.2. Generation of Non-KS MMPHs

To generate non-KS NBMMPHs, we make use of the following methods.

- **M1** consists of dropping vertices contained in single hyperedges (multiplicity m=1 ) [34] of either NBMMPHs or BMMPHs and a possible subsequent stripping of their hyperedges. The obtained smaller MMPHs are often non-KS, although never KS.
- **M2** consists of a random addition of hyperedges to MMPHs so as to obtain bigger ones, which then serve us to generate smaller non-KS NBMMPHs by stripping hyperedges randomly again;
- **M3** consists of the random deletion of vertices in either NBMMPHs or a BMMPHs until a non-KS NBMMPH is reached.

We combine all three methods to obtain an arbitrary number of non-KS NBMMPHs in an arbitrary dimension. The methods rely on the property of MMPHs where, by stripping an MMPH or NBMMPH (critical or not) or BMMPH of its hyperedges, we can arrive at smaller non-KS NBMMPHs in contrast to a critical KS NBMMPH whose stripping of hyperedges can never yield another (smaller) NBMMPH.

### 2.3. Dimensions Three to Five and the Three Classes of Non-KS Contextual Sets from the Literature

**Figure 1:**
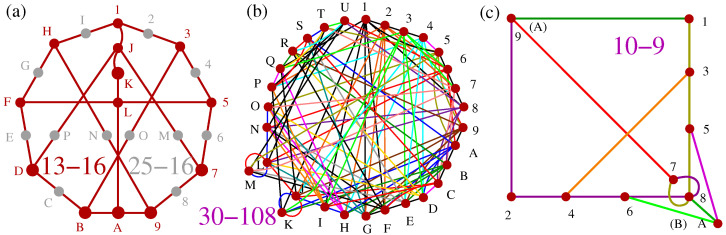
(**a**) Yu-Oh’s three-dim non-KS 13-16 non-KS NBMMPH ( [36] Figure 2); gray vertices that enlarge 13-16 to 25-16 are necessary for coordinatization and implementation; (**b**) Howard, Wallman, Veitech, and Emerson’s four-dim 30-108 non-KS NBMMPH ([4] Figure 2); (**c**) Cabello, Portillo, Solís, and Svozil’s five-dim 10-9 non-KS NBMMPH ([42] Figure 5a); the original symbols are presented in brackets (A,B).

In Figure 1, we give examples from each of the three classes of non-KS sets referred to in the Introduction. Here, we remind the reader that *k*-*l* MMPHs refer to hypergraphs with *k* vertices and *l* hyperedges (Definition 1), while the corresponding graphs have more than *l* edges. For example, in Figure 1a, the hypergraph hyperedge ALK corresponds to a graph clique with three edges: AL, LK, and KA.

Yu-Oh’s three-dim non-KS NBMMPH, shown in Figure 1a, is presumably the earliest of the kind. It is operator-based, but the operators are defined via states/vectors/vertices of 13-16 MMPH, as reviewed in [41]. Since orthogonal vectors in a three-dim space form triples, full representation requires 25-16, as indicated by the gray vertices in the figure, which can be obtained from Peres’ 33-40 [41] by stripping hyperedges and the 13-16 from it by removing m=1 vertices, i.e., via **M1**. The 13-16 MMPH is not critical, and it contains four critical sub-MMPHs, the smallest of which is 10-9 [41].

Howard, Wallman, Veitech, and Emerson’s four-dim 30-108 non-KS NBMMPH, shown in Figure 1b, which was obtained from the set of stabilizer states was used to prove that the underlying contextuality is essential for quantum computation. We discuss its filled 232-108 MMPH and its critical 24-71 MMPH in [34].

Cabello, Portillo, Solís, and Svozil’s five-dim 10-9 non-KS NBMMPH, shown in Figure 1b, is one of the minimal five-dim true-implies-false sets (TIFS) ([42] Figure 5a). It is not critical, and the only critical part it contains is a 10-7, but it is not a TIFS any more. The coordinatization of the filled 10-9 (31-9, which includes the coordinatization of 10-9 itself) can be built from the {0,±1,2} components and is given in Appendix A.

Our methods can generate NBMMPHs that are critical as well as those that are not. Therefore, although none of the aforementioned examples are critical, we focus on critical ones, because they offer the simplest implementation and presentation. The rationale for adopting such an approach is that only minimal contextual sets, i.e., critical NBMMPHs, are relevant for experimental implementations, since their supersets just contain additional orthogonalities that do not change the contextuality property of their smallest critical set. Hence, while designing MMPHs for particular implementations, we should attempt to find the ones that are critical and are provided via automated generations of MMPHs.

**Figure 2:**
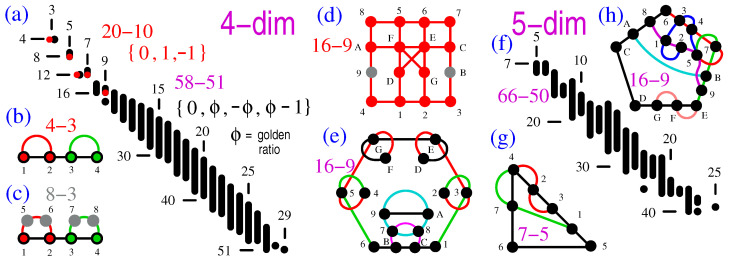
(**a**) Distributions of critical four-dim non-KS NBMMPHs obtained from submaster 20-10, which was obtained from (Peres’) 24-24 supermaster (generated by vector components {0,±1} ) by **M1** (dots in red) and from submaster 58-51, itself obtained from the 60-72 supermaster (generated by vector components {0,±ϕ,ϕ−1} , where ϕ is the golden ratio: $\frac{1+\sqrt{5}}{2}$ ) by **M1** (in black); the abscissa is *l* (number of hyperedges); and the ordinate is *k* (number of vertices). The dots represent (k,l) . Consecutive dots (same *l*) are shown as strips; (**b**) the smallest non-KS in the distributions: 4-3; (**c**) BMMPH 8-3—filled with 4-3—which one needs for obtaining the coordinatization and implementation of 4-3; (**d**) the 16-9 critical obtained from the 20-10 master; (**e**) the 16-9 critical obtained from the 58-51 master; (**f**) distributions of critical five-dim non-KS NBMMPHs obtained from submaster 66-50 which was obtained from the 105-136 supermaster (generated by vector components {0,±1} ); (**g**) the smallest critical; (**h**) a 16-9 critical for the sake of comparison with four-dim 16-9s; strings and coordinatizations are given in Appendix A.

**Figure 3:**
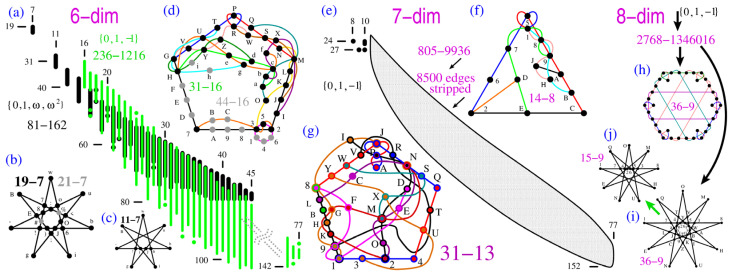
(**a**) Distributions of 6-dim critical non-KS NBMMPHs obtained from two different submasters—see text; (**b**) the smallest critical non-KS NBMMPH obtained from the former class by **M3**; it has a parity proof; (**c**) an even smaller critical non-KS NBMMPH obtained from it by hand; it has a parity proof; (**d**) the smallest critical non-KS NBMMPH obtained from the latter class by **M1**; (**e**) distributions of 7-dim critical non-KS NBMMPHs—see text; (**f**) 14-8 non-KS NBMMPH, one of the smallest non-KS NBMMPHs obtained via **M3** from the smallest KS NBMMPH 34-14; (**g**) 31-13 also obtained from the 34-14 (no m = 1 vertices essential for criticality); (**h**,**i**) two 8-dim KS MMPHs with the smallest number of hyperedges (9); (**i**) serves us in generating the 15-9 non-KS NBMMPH in (**j**); (**h**–**j**) MMPHs have parity proofs; strings and coordinatizations are given in Appendix A.

In [41], we give ample distributions of three-dim non-KS NBMMPHs obtained via **M1** and **M2**. Therefore, below, we give distributions and samples of just four- and five-dim critical non-KS NBMMPHs presented in Figure 2a,f. Here, we only point out that the KS “bug,” the 8-7 non-KS NBMMPH shown in ([41] Figure 3a), is the smallest three-dim non-KS NBMMPH that satisfies our requirement that at least one of the hyperedges must contain *n* vertices (*n* being the dimension of the considered MMPH), none of which has the multiplicity m=1 . Its string, the string of its filled MMPH, and their coordinatizations are given in Appendix A, as are the strings and coordinatizations of any other MMPH considered in the paper.

To obtain non-KS NBMMPHs via **M1**, we first generate the supermasters from the vector components. In the four-dim space, we obtain the 24-24 supermaster from the {0,±1} components and the 60-72 supermaster from the {0,±ϕ,ϕ−1} components, where $\phi =\frac{1+\sqrt{5}}{2}$ (the golden ratio). Their strings and coordinatizations are given in Appendix A. Then, we randomly strip hyperedges from them, e.g., 14 from 24-24 and 21 from the 60-72 supermaster, so as to obtain the 20-10 and 58-51 masters, respectively. From the latter masters, we remove m=1 vertices, and from any of them, we generate the classes of critical MMPHs by stripping them further until we obtain critical MMPHs that form the 20-10 and 58-51 non-KS classes. In the five-dim space, we obtain the 105-136 supermaster from the {0,±1} components. Its string and coordinatization are given in Appendix A. Further, we randomly strip 86 hyperedges to obtain a 66-50 master and eventually obtain its class of critical non-KS NBMMPHs.

We generate *n*-dim critical non-KS MMPHs under the requirement that at least one of their hyperedges must contain *n* vertices, of which none have a multiplicity of 1 ( m=1 ). (All examples from Figure 1 satisfy these conditions.) For instance, the smallest critical obtained in the four-dim distribution, shown in Figure 2a, is the 4-3 shown in Figure 2b, whose hyperedge 1234 is of such a kind. Its filled MMPH shown in Figure 2c provides a coordinatization necessary for the implementation of the 4-3. The 16-9 critical of the 20-10 master shown in Figure 2d contains two m=1 vertices (9,B), because m=1 vertices were stripped only once (from the master) when we started the generation of the 20-10 class. We can remove one or both of these vertices and still have a critical non-KS MMPH (15-9 or 14-9, respectively) if we want to for some reason. The 16-9 critical shown in Figure 2e has a parity proof, since in it, each vertex shares exactly two hyperedges, while there is an odd number of them (9). Strings and coordinatizations are given in Appendix A.

### 2.4. Dimensions Six to Eight

Cabello, Portillo, Solís, and Svozil also give a number of minimal six-dim TIFS non-KS NBMMPHs in ([42] Figure 7) along the same line as for their five-dim one shown in Figure 1c. To our knowledge, there are no explicit examples of non-KS NBMMPH in dimensions seven and eight in the literature. Therefore, we straightforwardly move to the generation of six- to eight-dim non-KS NBMMPHs.

An NBMMPH in the six-dim Hilbert space corresponds to a qubit entangled with a qutrit ( $H^{6}=H^{2}⊗H^{3}$ ) or a $\frac{5}{2}$ -spin system. So far, to obtain KS NBMMPH masters, the following vector components have been used: {0,±ω} , [44,52,53] ( ω is a cube root of 1, $\omega =e^{2\pi i/3}=\left(i\sqrt{3}-1\right)/2$ ), $\left\{0,\pm \omega ,\omega^{2}\right\}$ [53,54] and {0,±1} [52]. Since the first set of components yields a master with only three MMPHs, we make use of the other two to generate six-dim non-KS NBMMPHs.

The $\left\{0,1,\omega ,\omega^{2}\right\}$ set generates two unconnected masters: 591-1123 and 81-162 [53]. To obtain non-KS NBMMPHs, we apply **M1** to the 81-162 class. Their distribution is shown in Figure 3a in black. The {0,±1} set generates a 236-1216 master. Its non-KS NBMMPHs are also obtained via **M1** and are shown in Figure 3a in green.

In the seven-dim space, masters obtained from simple vector components, such as {0,±1} , are too big to be used for the exhaustive generation of a complete non-KS NBMMPH class. Instead, as in the previous six-dim case, we strip a significant portion of hyperedges from a master obtained from {0,±1} components and make use of the remaining MMPHs to obtain a non-KS class, as shown in Figure 3e; {0,±1} yields the 805-9936 master, and stripping of 8500 hyperedges leaves us with NBMMPHs with 436 hyperedge NBMMPHs, which generates a 436-hyperedge class. Since this class is still big, we have to repeat **M1** several times to obtain small non-KS critical NBMMPHs. As a result, hyperedges of all small NBMMPHs may contain some m=1 vertices essential for criticality, as shown in Figure 3f (the removal of vertex 6 would terminate the criticality of the MMPH). In dimensions greater than nine, such vertices do not appear, although even here we can avoid their generation by applying **M3** to KS NBMMPHs, as shown in Figure 3g.

The eight-dim MMPH master is big (2768-1346016), but the stripping technique can still provide us with non-KS NBMMPHs via **M1**. However, the MMPHs with m=1 vertices are also big, and obtaining small criticals with up to 40 hyperedges would require roughly one week on a supercomputer with 200 2.5 GHz CPUs working in parallel. We may be able to work around this problem by exploiting previously generated small KS criticals [52] so as to use them as masters for non-KS MMPHs while applying **M3**, as shown in Figure 3h–j (cf. the six-dim star in Figure 3b). Notice the graphical similarity of the four-dim 18-9 ([51] Figure 3a) and eight-dim 36-9 (shown in Figure 3h) for each vertex from the 18-9 vs. a pair of vertices in the 36-9. Since the distribution of eight-dim KS MMPHs in Ref. [52] is abundant, we can arbitrarily generate many non-KS NBMMPHs in this manner via **M3**.

### 2.5. Dimensions Nine to Eleven

**Figure 4:**
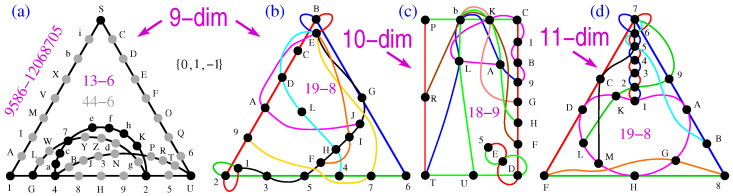
(**a**) The 44-6 BMMPH and its critical subgraph 13-6 non-KS NBMMPH directly obtained from the supermaster via **M1**; (**b**) the critical nine-dim 19-8 obtained via **M3** from the master 47-16; (**c**) the critical ten-dim 18-9 non-KS NBMMPH obtained via **M3** from the 50-15 master; (**d**) the critical eleven-dim 19-8 non-KS NBMMPH obtained via **M3** from the 50-14 master. Strings and coordinatizations are given in Appendix A.

The nine-dim NBMMPH master obtained from {0,±1} has 9586 vertices and 12,068,705 hyperedges and that is too big for the direct generation of critical MMPHs (via stripping and filtering), especially for higher dimensions. However, billions of BMMPHs can be generated from the master, and as we have already stressed, stripping them of m=1 often provides us with NBMMPHs. This renders **M1** applicable. Thus, after the random stripping of 12,068,200 hyperedges, we obtained submasters with 505 hyperedges. By requiring that at least one of the hyperedges contains *n* vertices and that some of them can have the multiplicity m=1 , our program STATES01 yields a series of critical NBMMPHs, the smallest of which is 13-6, as shown in Figure 4a. The hyperedge 4ac7efhK2 contains nine vertices. (Notice also that the 13-6 NBMMPH remains a critical non-KS NBMMPH with any, some, or all of a,c,e,f,h,K removed.) The filled 13-16, i.e., 44-6, also shown in Figure 4a, obtains the coordinatization directly from the supermaster, since the programs preserve the names of the vertices in the process of stripping and yielding sub-MMPHs. Obtaining a coordinatization via VECFIND takes too many CPU hours. The latter feature also makes **M2** inapplicable.

If we wanted to keep our *n*-vertex requirement in full (“no m=1 vertices”), in order to obtain critical non-KS NBMMPHs, we would need to employ **M3**, so as to apply it on KS NBMMPHs obtained via dimensional upscaling [55,56], as follows. We removed several vertices from the smallest critical 47-16 obtained in [56] until it was not critical any more. Then, STATES01 yielded the 19-8 critical shown in Figure 4b. (The removal of vertex L would terminate the criticality of the MMPH as with the seven-dim one shown in Figure 3f, but that would not affect the full *n*-vertex requirement.)

A 10-dim or any higher-dimensional masters are too big to be generated from vector components. Therefore, to obtain the non-KS MMPH in those dimensions, we rely on minimal KS NBMMPHs obtained via dimensional upscaling [56] while applying **M3**. The procedure consists of removing vertices and/or hyperedges in such a way that an NBMMPH stops being critical, which enables us to generate smaller critical non-KS NBMMPHs from it via STATES01.

In Figure 4c, we show an 18-9 10-dim critical obtained via this approach from the 50-15 KS MMPH master [56].

In Figure 4d, we show a 19-8 11-dim critical obtained via the same approach from the 50-14 KS MMPH master [56].

In the following sections, we stay with this approach while applying **M3**.

### 2.6. Dimensions 12 to 16

**Figure 5:**
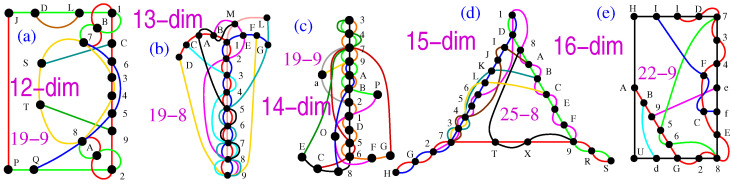
(**a**) Twelve-dim 19-9 critical non-KS NBMMPH directly obtained from the master 52-9 via **M3**; (**b**) 13-dim 19-8 critical non-KS NBMMPH obtained from the master 63-16, where the hyperedges do not form any loop with an order of three or higher; (**c**) 14-dim critical obtained from 66-15, where the maximal loop also has an order of 2; (**d**) 15-dim 25-8 critical from the 66-14 master; (**e**) 16-dim 22-9 critical from the 70-9 master, where all criticals are obtained via **M3**; all criticals and masters are given in the Appendix A.

It has been proven that the minimal complexity (minimal number of hyperedges or vertices) of the dimensional upscaling of KS MMPHs does not scale up with dimension [55]. In  [56], we give a constructive proof that the minimal number of hyperedges of KS MMPHs repeatedly fluctuates between nine and sixteen, which confirms this result. In the previous section we provide constructive generations of critical non-KS NBMMPHs in dimensions nine to eleven and in this section, in Figure 5a–e in dimensions twelve to sixteen, whose minimal number of hyperedges fluctuates between eight (odd dimensions) and nine (even dimensions) under the requirement that at least one the hyperedges contains *n* vertices, none of which has the multiplicity m=1 . In lower dimensions (3–6), the minimal number of hyperedges is even smaller.

## 3. Discussion

In this paper, we first generated non-KS contextual NBMMPHs (non-binary MMP hypergraphs) with the help of master sets generated from simple vector components whose complexity exponentially scales with dimension—for dimensions four to eight—and then by means of methods whose complexity does not scale with dimension. The need for developing such methods and obtaining MMPHs in higher dimensions has emerged from recent elaborations of classes of contextual sets that are not of the KS kind, all of which have an MMP hypergraph representation. Examples of such elaborations in the literature and their correspondence with MMPHs are given in Section 2.3. In subsequent sections, we presented generations of non-KS NBMMPHs in spaces of up to 16-dim.

In Section 2.1, we presented the formalism and language of MMPH, and in Section 2.2, we presented the methods of generating them. In Section 2.3, we reviewed the most prominent examples of non-KS sets from the literature in dimensions three to five, represented them via MMPH formalism, and generated several new non-KS MMPHs in dimensions four and five with several coordinatizations. In Section 2.3, we then went up to the eight-dim spaces and showed that the arbitrarily exhaustive generation of MMPHs gets more and more computationally demanding from three-dim to eight-dim spaces due to the exponentially increasing size of the MMPH masters obtained from vector components and the exponential complexity of extracting of NBMMPH classes from them. This is exacerbated by the ratio of NBMMPHs and BMMPHs, which starts with less than 0.1% in four-dim spaces and grows exponentially with the dimension. So, in the nine-dim space in Section 2.5 with a master containing 9586 vertices and 12,068,705 hyperedges, we can strip any number of hyperedges from the master, but the probability of finding any NBMMPH among the obtained MMPHs decreases with size (e.g., searching for them in MMPHs with more than a few thousand hyperedges would take “forever” for any practical purpose). In spaces with dimensions of 10 and greaterm no method for obtaining MMPH masters from vector components is available anymore.

Therefore, to ensure arbitrarily exhaustive generation of MMPHs in ever higher dimensions, we need a method whose complexity does not grow with the dimensions. For comparatively small KS MMPHs, such a method—dimensional upscaling—was recently developed in [56] based on previous results in [55]. In this paper, we put forward a method of generating non-KS NBMMPHs whose complexity also does not scale up with the dimensions and which makes use of KS MMPHs obtained by the former KS method (in Section 2.5 and Section 2.6). The method applies to the generation of comparatively small MMPHs that are still suitable for any practical implementation since we can always obtain bigger MMPHs at the cost of the time a generation would take and since really big MMPHs cannot be generated at all, and even if they could, they would be unimplementable. The minimal complexity (minimal number of hyperedges or vertices) of KS MMPHs repeatedly fluctuates between nine and sixteen, while for non-KS NBMMPHs, it fluctuates between eight (odd dimensions) and nine (even dimensions) in seven- to sixteen-dim spaces. In three- to six-dim, it even goes down to three. We provide a list of them in Table 1.

## 4. Methods

The methods used to handle quantum contextual sets rely on algorithms and programs within the MMP language: VECFIND, STATES01, MMPSTRIP, MMPSHUFFLE, SUBGRAPH, LOOP, and SHORTD developed in [8,47,48,49,50,51,57,58]. They are freely available at http://puh.srce.hr/s/Qegixzz2BdjYwFL (accessed on 22 July 2023). MMPHs can be visualized via hypergraph figures consisting of dots and lines and represented as a string of ASCII characters. The latter representation enables the processing of billions of MMPHs simultaneously via supercomputers and clusters. For the latter elaboration, we developed other dynamical programs specifically to handle and parallelize jobs with arbitrary numbers of MMP hypergraph vertices and edges.

## 5. Conclusions

To summarize, based on elaborations of non-KS sets that recently appeared in the literature and of which we provided several examples in Section 2.3, we developed methods of generating comparatively small non-KS contextual sets in high-dimensional spaces whose complexity does not grow with the number of dimensions. We provided examples in all dimensions up to 16. A more detailed summary of the achieved results is given in Section 3.

## Figures and Tables

**Table 1:** The smallest critical non-KS MMPHs obtained via the small vector component method and by the dimensional upscaling method via **M1** and **M3**. Notice the steady fluctuation in the number of hyperedges over dimensions which is consistent with our previous result showing that the minimum complexity of NBMMPHs does not grow with the dimensions. The MMPH strings and coordinatizations of both the criticals and their masters are given in Appendix A. ϕ is the Golden ratio, and ω is the cube root of 1.

dim	Smallest Critical MMPHs	Master	Vector Components
3-dim	8-7 (Kochen–Specker’s “bug”)	49-36 (Bub’s KS MMPH)	{0,±1,±2,5}
4-dim	4-3	8-3	{0,±1}
4-dim	16-9	58-51	{0,±ϕ,ϕ−1}
5-dim	7-5	16-5	{0,±1}
6-dim	11-7	19-7	$\left\{0,1,\omega ,\omega^{2}\right\}$
7-dim	14-8	34-14	{0,±1}
8-dim	15-9	2768-1346016	{0,±1}
9-dim	13-6	9586-12068705	{0,±1}
9-dim	19-8	47-16	{0,±1}
10-dim	18-9	50-15	{0,±1}
11-dim	19-8	50-14	{0,±1}
12-dim	19-9	52-9	{0,±1}
13-dim	19-8	63-16	{0,±1}
14-dim	19-9	66-15	{0,±1}
15-dim	25-8	66-14	{0,±1}
16-dim	22-9	70-9	{0,±1}

## Data Availability

Additional data are available from the author on request.

## Abbreviations

   The following abbreviations are used in this manuscript:

MMPH	McKay–Megill–Pavičić hypergraph (Definition 1)
NBMMPH	Non-binary McKay–Megill–Pavičić hypergraph (Definition 2)
BMMPH	Binary McKay–Megill–Pavičić hypergraph (Definition 5)
KS	Kochen–Specker (Definition 3)
non-KS	Non-Kochen–Specker (Definition 4)
**M1, M2, M3**	Methods 1,2,3 (Section 2.2)

## Appendix A. SCII Strings of Non-KS MMPH Classes and Their Masters and Supermasters

Below, we give strings and coordinatizations of all MMPHs referred to in the main body of the paper. The first hyperedges in a line of a critical NBMMPH often correspond to the biggest loops in the figures.

### Appendix A.1. Three-dim MMPHs

**8-7** (KS “bug”) 123,34,45,567,78,81,26.

**13-7** (filled **8-7**) 123,394,4A5,567,7B8,8C1,2D6. 1=(0,0,1), 2=(0,1,0), 3=(1,0,0), 4=(0,1,1), 5=(1,1,−1), 6=(1,0,1), 7=(−1,2,1), 8=(2,1,0), 9=(0,1,−1), A=(2,−1,1), B=(1,−2,5), C=(1,−2,0), D=(1,0,−1)

### Appendix A.2. Four-dim MMPHs

**4-3** 12,34,1234.

**8-3** (filled **4-3**) 1562,3784,1234. 1=(0,0,0,1), 2=(0,0,1,0), 3=(0,1,0,0), 4=(1,0,0,0), 5=(1,1,0,0), 6=(1,−1,0,0), 7=(0,0,1,1), 8=(0,0,1,−1)

**16-9**   3124,49A8,8567,7BC3,DE,FG,GE62,FD51,FECA.

**20-9** (filled **16-9**) 1=(1,0,0,−1), 2=(0,1,1,0), 3=(1,1,−1,1), 4=(1,−1,1,1), 5=(1,0,0,1), 6=(0,1,−1,0), 7=(1,1,1,−1), 8=(−1,1,1,1), 9=(0,0,1,−1), A=(1,1,0,0), B=(0,0,1,1), C=(1,−1,0,0), D=(0,1,0,0), E=(0,0,0,1), F=(0,0,1,0), G=(1,0,0,0), H=(1,0,1,0), I=(1,0,−1,0), J=(0,1,0,1), K=(0,1,0,−1)

**24-24** (Peres’ supermaster) LMNO,HIJK,DEFG,BCFG,9ADE,78EG,56DF,5678,9ABC,68JK,57HI,ACIK,9BHJ,1234,4DGO,3EFN,258M,167L,19CM,2ABL,3HKO,4IJN,34NO,12LM. 1=(0,0,0,1), 2=(0,0,1,0), 3=(1,1,0,0), 4=(1,−1,0,0), 5=(0,1,0,−1), 6=(1,0,−1,0), 7=(1,0,1,0), 8=(0,1,0,1), 9=(0,1,−1,0), A=(1,0,0,−1), B=(1,0,0,1), C=(0,1,1,0), D=(1,1,1,1), E=(1,−1,−1,1), F=(1,−1,1,−1), G=(1,1,−1,−1), H=(−1,1,1,1), I=(1,1,−1,1), J=(1,1,1,−1), K=(1,−1,1,1), L=(0,1,0,0), M=(1,0,0,0), N=(0,0,1,1), O=(0,0,1,−1)

**16-9**       231,1BC6,654,45GF,FGED,DE32,789A,7BC8,9A.

**20-9** (filled **16-9**) 2H31,1BC6,6I54,45GF,FGED,DE32,789A,7BC8,9JKA. 1=(0,0,0, ϕ ), 2=( ϕ −1,0,− ϕ ,0), 3=( ϕ ,0, ϕ −1,0), 4=(0, ϕ ,0, ϕ ), 5=(0, ϕ ,0,− ϕ ), 6=(0,0, ϕ ,0), 7=(0,0, ϕ , ϕ ), 8=(0,0, ϕ ,− ϕ ), 9=( ϕ , ϕ −1,0,0), A=( ϕ −1,− ϕ ,0,0), B=( ϕ , ϕ ,0,0), C=( ϕ ,− ϕ ,0,0), D=(0, ϕ −1,0,− ϕ ), E=(0, ϕ ,0, ϕ −1), F=( ϕ ,0, ϕ ,0), G=( ϕ ,0,− ϕ ,0), H=(0, ϕ ,0,0), I=( ϕ ,0,0,0), J=(0,0, ϕ , ϕ −1), K=(0,0, ϕ −1,− ϕ )

**60-72** (supermaster) 1234,1256,1278,129A,13BC,13DE,13FG,1HI4,1JK4,1LM4,23NO,23PQ,23RS,2TU4,2VW4,2XY4,Za34,Za56,Za78,Za9A,Z5bc,Zde6,fg34,fg56,fg78,fg9A,hi34,hi56,hi78,hi9A,ajk6,a5lm,ano6,apq6,TUBC,TUDE,TUFG,TBbo,TCmd,VWBC,VWDE,VWFG,XYBC,XYDE,XYFG,HINO,HIPQ,HIRS,HNco,HOme,JKNO,JKPQ,JKRS,LMNO,LMPQ,LMRS,UBle,UnCc,UrsC,UtCu,jkbc,INld,InOb,IvwO,IxOy,nbco,rsbo,pbcq,vwco,tbuo,xyco,lmde. 1=(0,0,0, ϕ ), 2=(0,0, ϕ ,0), 3=(0, ϕ ,0,0), 4=( ϕ ,0,0,0), 5=( ϕ , ϕ ,0,0), 6=( ϕ ,− ϕ ,0,0), 7=( ϕ , ϕ −1,0,0), 8=( ϕ −1,− ϕ ,0,0), 9=(− ϕ , ϕ −1,0,0), A=( ϕ −1, ϕ ,0,0), B=( ϕ ,0, ϕ ,0), C=( ϕ ,0,− ϕ ,0), D=(− ϕ ,0, ϕ −1,0), E=( ϕ −1,0, ϕ ,0), F=( ϕ ,0, ϕ −1,0), G=( ϕ −1,0,− ϕ ,0), H=(0, ϕ , ϕ ,0), I=(0, ϕ ,− ϕ ,0), J=(0, ϕ −1, ϕ ,0), K=(0,− ϕ , ϕ −1,0), L=(0, ϕ −1,− ϕ ,0), M=(0, ϕ , ϕ −1,0), N=( ϕ ,0,0, ϕ ), O=( ϕ ,0,0,− ϕ ), P=( ϕ ,0,0, ϕ −1), Q=( ϕ −1,0,0,− ϕ ), R=(− ϕ ,0,0, ϕ −1), S=( ϕ −1,0,0, ϕ ), T=(0, ϕ ,0, ϕ ), U=(0, ϕ ,0,− ϕ ), V=(0, ϕ ,0, ϕ −1), W=(0, ϕ −1,0,− ϕ ), X=(0,− ϕ ,0, ϕ −1), Y=(0, ϕ −1,0, ϕ ), Z=(0,0, ϕ , ϕ ), a=(0,0, ϕ ,− ϕ ), b=( ϕ ,− ϕ ,− ϕ , ϕ ), c=( ϕ ,− ϕ , ϕ ,− ϕ ), d=( ϕ , ϕ , ϕ ,− ϕ ), e=( ϕ , ϕ ,− ϕ , ϕ ), f=(0,0, ϕ , ϕ −1), g=(0,0, ϕ −1,− ϕ ), h=(0,0,− ϕ , ϕ −1), i=(0,0, ϕ −1, ϕ ), j=( ϕ , ϕ , ϕ −1, ϕ −1), k=( ϕ −1, ϕ −1,− ϕ ,− ϕ ), l=(− ϕ , ϕ , ϕ , ϕ ), m=( ϕ ,− ϕ , ϕ , ϕ ), n=( ϕ , ϕ , ϕ , ϕ ), o=( ϕ , ϕ ,− ϕ ,− ϕ ), p=(− ϕ ,− ϕ , ϕ −1, ϕ −1), q=( ϕ −1, ϕ −1, ϕ , ϕ ), r=( ϕ , ϕ −1, ϕ , ϕ −1), s=( ϕ −1,− ϕ , ϕ −1,− ϕ ), t=(− ϕ , ϕ −1,− ϕ , ϕ −1), u=( ϕ −1, ϕ , ϕ −1, ϕ ), v=( ϕ , ϕ −1, ϕ −1, ϕ ), w=( ϕ −1,− ϕ ,− ϕ , ϕ −1), x=(− ϕ , ϕ −1, ϕ −1,− ϕ ), y=( ϕ −1, ϕ , ϕ , ϕ −1)

### Appendix A.3. Five-dim MMPHs

**7-5** 41235,56,674,234,714.

**16-5** (**7-5** filled) 41235,589A6,6BC74,2DE34,7FG14. 1=(0,0,1,0,0), 2=(1,−1,0,0,0), 3=(1,1,0,0,0), 4=(0,0,0,0,1), 5=(0,0,0,1,0), 6=(0,1,1,0,0), 7=(1,0,0,1,0), 8=(1,0,0,0,−1), 9=(0,1,−1,0,0), A=(1,0,0,0,1), B=(1,−1,1,−1,0), C=(1,1,−1,−1,0), D=(0,0,1,1,0), E=(0,0,1,−1,0), F=(0,1,0,0,0), G=(1,0,0,−1,0)

**16-9**    63457,75B9E,EFGD,DC,CA86,12345,89125,AB,FGE.

**26-9** (**16-9** filled) 63457,75B9E,EFHGD,DIJKC,CAL86,12345,89125,AMNOB,FPQGE. 1=(1,1,1,−1,0), 2=(1,1,−1,1,0), 3=(1,−1,1,1,0), 4=(−1,1,1,1,0), 5=(0,0,0,0,1), 6=(0,0,1,−1,0), 7=(1,1,0,0,0), 8=(0,0,1,1,0), 9=(1,−1,0,0,0), A=(0,1,0,0,1), B=(0,0,1,0,0), C=(1,0,0,0,0), D=(0,1,1,0,0), E=(0,0,0,1,0), F=(1,0,0,0,1), G=(0,1,−1,0,0), H=(1,0,0,0,−1), I=(0,0,0,1,1), J=(0,1,−1,1,−1), K=(0,1,−1,−1,1), L=(0,1,0,0,−1), M=(1,1,0,−1,−1), N=(1,−1,0,−1,1), O=(1,0,0,1,0), P=(1,1,1,0,−1), Q=(−1,1,1,0,1)

**105-136** (supermaster) 12345,12367,12489,12AB5,134CD,13EF5,1GH45,1GH67,1G6IJ,1GKL7,1H6MN,1HOP7,1EF89,1E8IP,1E9KN,1F8ML,1F9OJ,1ABCD,1ACJP,1ADLN,1QRST,1QUVW,1XYSZ,1XabW,1BCMK,1BDOI,1cYVd,1caeT,1fRbd,1fUeZ,1OIJP,1MKLN,234gh,23ij5,2kl45,2kl67,2k6mn,2kop7,2l6qr,2lst7,2ij89,2i8mt,2i9or,2j8qp,2j9sn,2ABgh,2Agtn,2Ahpr,2uvSw,2uxyW,2z!S",2z#$W, 2Bgoq,2Bhsm,2%!yd,2%#ew,2&v$d,2&xe",2smtn,2oqpr,’(345,’(367,’(489,’(AB5,’36)*,’3-/7, ’48:;,’4<=9,’A>?5,’@[B5,(36\],(3^_7,(48‘{,(4|}9,(A∼+15,(+2+3B5,3ijCD,3iC_*,3iD-\,3jC/],3jD^),3EFgh,3Eg_),3Eh/\,3+4+5Vw,3+4+6yT,3+7+8V",3+7+9$T,3Fg-],3Fh^*,3+A+8yZ, 3+A+9bw,3+B+5$Z,3+B+6b",3^_)*,3-/\],kl4CD,klEF5,k4C};,k4D<‘,kE+3?5,kF@∼5, l4C={,l4D|:, lE[+15,lF+2>5,GH4gh,GHij5,G4g}:,G4h=‘,Gi+3>5,Gj[∼5,+C4+5Rx,+C4+6vU,+C+7X%5,+C+Azc5, +D4+8R#,+D4+9!U,+D+4X&5,+D+Buc5,H4g<{,H4h|;,Hi@+15, Hj+2?5,+E4+8va,+E4+9Yx,+E+4zf5, +E+BQ%5,+F4+5!a,+F4+6Y#,+F+7uf5,+F+AQ&5,4|}:;,4<=‘{,+2+3>?5,@[∼+15. 1=(0,0,0,0,1), 2=(0,0,0,1,0), ’=(0,0,0,1,1), (=(0,0,0,1,−1), 3=(0,0,1,0,0), k=(0,0,1,0,1), l=(0,0,1,0,−1), G=(0,0,1,1,0), +C=(0,0,1,1,1), +D=(0,0,1,1,−1), H=(0,0,1,−1,0), +E=(0,0,1,−1,1), +F=(0,0,−1,1,1), 4=(0,1,0,0,0), i=(0,1,0,0,1), j=(0,1,0,0,−1), E=(0,1,0,1,0), +4=(0,1,0,1,1), +7=(0,1,0,1,−1), F=(0,1,0,−1,0), +A=(0,1,0,−1,1), +B=(0,−1,0,1,1), A=(0,1,1,0,0), u=(0,1,1,0,1), z=(0,1,1,0,−1), Q=(0,1,1,1,0), +2=(0,1,1,1,1), @=(0,1,1,1,−1), X=(0,1,1,−1,0), [=(0,1,1,−1,1), +3=(0,1,1,−1,−1), B=(0,1,−1,0,0), %=(0,1,−1,0,1), &=(0,−1,1,0,1), c=(0,1,−1,1,0), ∼=(0,1,−1,1,1), >=(0,1,−1,1,−1), f=(0,−1,1,1,0), ?=(0,1,−1,−1,1), +1=(0,−1,1,1,1), 5=(1,0,0,0,0), g=(1,0,0,0,1), h=(1,0,0,0,−1), C=(1,0,0,1,0), +8=(1,0,0,1,1), +5=(1,0,0,1,−1), D=(1,0,0,−1,0), +6=(1,0,0,−1,1), +9=(−1,0,0,1,1), 8=(1,0,1,0,0), !=(1,0,1,0,1), v=(1,0,1,0,−1), Y=(1,0,1,1,0), |=(1,0,1,1,1), <=(1,0,1,1,−1), R=(1,0,1,−1,0), ==(1,0,1,−1,1), }=(1,0,1,−1,−1), 9=(1,0,−1,0,0), x=(1,0,−1,0,1), #=(−1,0,1,0,1), U=(1,0,−1,1,0), ‘=(1,0,−1,1,1), :=(1,0,−1,1,−1), a=(−1,0,1,1,0), ;=(1,0,−1,−1,1), {=(−1,0,1,1,1), 6=(1,1,0,0,0), $=(1,1,0,0,1), y=(1,1,0,0,−1), b=(1,1,0,1,0), ^=(1,1,0,1,1), −=(1,1,0,1,−1), V=(1,1,0,−1,0), /=(1,1,0,−1,1), _=(1,1,0,−1,−1), e=(1,1,1,0,0), s=(1,1,1,0,1), o=(1,1,1,0,−1), O=(1,1,1,1,0), M=(−1,1,1,1,0), I=(1,−1,−1,1,0), K=(1,1,1,−1,0), q=(−1,1,1,0,1), m=(1,−1,−1,0,1), S=(1,1,−1,0,0), p=(1,1,−1,0,1), t=(1,1,−1,0,−1), L=(1,1,−1,1,0), d=(−1,1,1,0,0), J=(1,−1,1,−1,0), P=(1,1,−1,−1,0), N=(1,−1,1,1,0), n=(1,−1,1,0,−1), 7=(1,−1,0,0,0), w=(1,−1,0,0,1), "=(−1,1,0,0,1), T=(1,−1,0,1,0), \=(1,−1,0,1,1), )=(1,−1,0,1,−1), Z=(−1,1,0,1,0), *=(1,−1,0,−1,1), ]=(−1,1,0,1,1), W=(1,−1,1,0,0), r=(1,−1,1,0,1)

**10-9** ([42] Figure 5a) 17835,27846,91,97,92,8A,34,6A,5A.

**31-9** (**10-9** filled) 17835,27846,9BCD1,9EFG7,9HIJ2,8KLMA,3NOP4,6QRSA,5TUVA. 1=(0,0,1,0,−1), 2=(0,0,0,1,−1), 3=(0,0,0,1,0), 4=(0,0,1,0,0), 5=(0,0,1,0,1), 6=(0,0,0,1,1), 7=(0,1,0,0,0), 8=(1,0,0,0,0), 9=(0,0,1,1,1), A=(0,1,1,1,−1), B=(0,0,−1,2,−1), C=(1,2,0,0,0), D=(2,−1,0,0,0), E=(0,0,−1,−1,2), F=(1,0,1,−1,0), G=(2,0,−1,1,0), H=(0,0,2,−1,−1), I=(1,1,0,0,0), J=(1,−1,0,0,0), K=(0,0,1,−1,0), L=(0,1,0,0,1), M=(0,−1,1,1,1), N=(0,0,0,0,1), O=(−1,2,0,0,0), P=(2,1,0,0,0), Q=(0,1,1,−1,1), R=(1,1,−1,0,0), S=(2,−1,1,0,0), T=(0,1,0,−1,0), U=(2,1,−1,1,1), V=(2,−1,1,−1,−1)

### Appendix A.4. Six-dim MMPHs

**19-7**       7!8gw,woO6i,i;)EB,B<:b7,’!)Jb6,’o:8Eu,;<OJgu.

**11-7**       w!g,wi6,’!)Jb6,’u,B)i,Jgu,Bb.

**21-7** (**11-7, 19-7** filled) w!78#g,woOi6%,’!)Jb6,’o:8Eu,;B)i#E,;<OJgu,B<7:b%.w=( ω ,1,1, $\omega^{2}$ ,1, ω ), ’=( $\omega^{2}$ , ω , ω ,1,1,1), ;=( ω ,1,1, $\omega^{2}$ , ω ,1), !=( ω ,1, $\omega^{2}$ ,1, ω ,1), B=(1, $\omega^{2}$ , ω ,1, ω ,1), <=( ω ,1, ω ,1,1, $\omega^{2}$ ), o=(1, $\omega^{2}$ ,1,1,1, $\omega^{2}$ ), )=( ω , $\omega^{2}$ ,1,1,1, ω ), 7=(1, ω , $\omega^{2}$ ,1,1, ω ), O=(1, ω , ω , $\omega^{2}$ ,1,1), :=( $\omega^{2}$ ,1,1,1, ω , ω ), 8=(1, ω ,1, $\omega^{2}$ , ω ,1), i=( $\omega^{2}$ ,1, $\omega^{2}$ ,1,1,1), J=(1, ω ,1,1, ω , $\omega^{2}$ ), #=(1,1,1,1, $\omega^{2}$ , $\omega^{2}$ ), b=(1,1,1, ω ,1,1), 6=(1,1, ω ,1, $\omega^{2}$ , ω ), g=( $\omega^{2}$ , $\omega^{2}$ ,1,1,1,1), E=(1,1, ω , $\omega^{2}$ ,1, ω ), %=( ω , ω ,1,1, $\omega^{2}$ ,1), u=(1,1, $\omega^{2}$ ,1, $\omega^{2}$ ,1)

**79-162** (81-162 stripped; %,#)) 123456,12789A,1BCD5E,1B7FGH,1ICJ9K,1I3LGM,1NODAP,1NQ4HR, 1SOJ6T,1SU8MR,1VQLET,1VUFKP,WXY45Z,WX7abA,Wcde5E,Wc7Ffg,WIdJbh,WIYifM,WjkeAP,WjQ4gl,WSkJZm,WSnaMl,WoQiEm,WonFhP,pqY89Z,pq3ab6,pcre9K,pc3Lsg,pBrDbh,pBYisH,pjte6T,pjU8gu,pNtDZm,pNnaHu,pvnLhT,pvUiKm,wxyD5Z,wx7azH,w!"e56,w!78g,wI"Lzh,wIyiK,w$keHR,w$ODgl,wVkLZ,wV&aKl,woOi6,wo&8hR,’(yDbA,’(Y4zH,’!)Jb6,’!Y8*M,’c)LzE,’cyF*K,’-kJHu,’-tDMl,’vkLA/,’v:4Kl,’otF6/,’o:8Eu,;("e9A,;(34g,;x)J9Z,;x3a*M,;B)iE,;B"F*h,;<teMR,;<OJgu,;vOiA=,;v>4hR,;VtFZ=,;V>aEu,?!reGM,?!CJsg,?qyFGZ,?qCazE,?2yisA,?2r4zh,?$:eET,?$UFg/,?-&JhT,?-UiM,?N:4Z,?N&aA/,@(deGH,@(CDfg,@X)LGZ,@XCa*K,@2)if6,@2d8*h,@<:eKP,@<QLg/,@->DhP,@-QiH=,@S:8Z=,@S>a6/,[xdJsH,[xrDfM,[X"LsA,[Xr4K,[q"Ff6,[qd8E,[<&JKm,[<nLM,[$>DEm,[$nFH=,[j>46,[j&8A=,(S"Uzm,(SynT,(VdUb,(VY&fT,(oCn9,(o3&Gm,xj)UzP,xjyQ*T,xvdU5/,xv7:fT,xorQ9/,xo3:sP,!N)nP,!N"Q*m,!vCn5=,!v7>Gm,!VrQb=,!VY>sP,X$)UbR,X$YO*T, X-"U5u,X-7tT,XorOGu,XoCtsR,q<yQbR,q<YOzP,q-"Q9l,q-3kP,qvdOGl,qvCkfR,2<yn5u,2<7tzm, 2$)n9l,2$3k*m,2Vdtsl,2Vrkfu,c-3&5=,c-7>9,cN)&fR,cNdO*,cSy>sR,cSrOz=,B<Y&5/,B<7:b,Bj)&Gl,BjCk*,BS":sl,BSrk/,I$Y>9/,I$3:b=,Ijy>Gu,IjCtz=,IN":fu,INdt/.

**81-162** 123456,12789A,1BCD5E,1B7FGH,1ICJ9K,1I3LGM,1NODAP,1NQ4HR,1SOJ6T,1SU8MR,1VQLET,1VUFKP,WXY45Z,WX7abA,Wcde5E,Wc7Ffg,WIdJbh,WIYifM,WjkeAP,WjQ4gl,WSkJZm,WSnaMl,WoQiEm,WonFhP,pqY89Z,pq3ab6,pcre9K,pc3Lsg,pBrDbh,pBYisH,pjte6T,pjU8gu,pNtDZm,pNnaHu,pvnLhT,pvUiKm,wxyD5Z,wx7azH,w!"e56,w!78#g,wI"Lzh,wIyi#K,w$keHR,w$ODgl,wVkLZ%,wV&aKl,woOi6%,wo&8hR,’(yDbA,’(Y4zH,’!)Jb6,’!Y8*M,’c)LzE,’cyF*K,’-kJHu,’-tDMl,’vkLA/,’v:4Kl,’otF6/,’o:8Eu,;("e9A,;(34#g,;x)J9Z,;x3a*M,;B)i#E,;B"F*h,;<teMR,;<OJgu,;vOiA=,;v>4hR,;VtFZ=,;V>aEu,?!reGM,?!CJsg,?qyFGZ,?qCazE,?2yisA,?2r4zh,?$:eET,?$UFg/,?-&JhT,?-UiM%,?N:4Z%,?N&aA/,@(deGH,@(CDfg,@X)LGZ,@XCa*K,@2)if6,@2d8*h,@<:eKP,@<QLg/,@->DhP, @-QiH=,@S:8Z=,@S>a6/,[xdJsH,[xrDfM,[X"LsA,[Xr4#K,[q"Ff6,[qd8#E,[<&JKm,[<nLM%, [$>DEm,[$nFH=,[j>46%,[j&8A=,(S"Uzm,(Syn#T,(VdUb%,(VY&fT,(oCn9%,(o3&Gm,xj)UzP,xjyQ*T, xvdU5/,xv7:fT,xorQ9/,xo3:sP,!N)n#P,!N"Q*m,!vCn5=,!v7>Gm,!VrQb=,!VY>sP,X$)UbR,X$YO*T,X-"U5u,X-7t#T,XorOGu,XoCtsR,q<yQbR,q<YOzP,q-"Q9l,q-3k#P,qvdOGl,qvCkfR,2<yn5u,2<7tzm,2$)n9l,2$3k*m,2Vdtsl,2Vrkfu,c-3&5=,c-7>9%,cN)&fR,cNdO*%,cSy>sR,cSrOz=,B<Y&5/,B<7:b%,Bj)&Gl,BjCk*%,BS":sl,BSrk#/,I$Y>9/,I$3:b=,Ijy>Gu,IjCtz=,IN":fu,INdt#/. 1=( ω ,1,1,1,1,1), W=( ω ,1,1,1, $\omega^{2}$ , ω ),p=( ω ,1,1,1, ω , $\omega^{2}$ ), w=( ω ,1,1, $\omega^{2}$ ,1, ω ),’=( $\omega^{2}$ , ω , ω ,1,1,1), ;=( ω ,1,1, $\omega^{2}$ , ω ,1), ?=( ω ,1,1, ω ,1, $\omega^{2}$ ), @=( ω ,1,1, ω , $\omega^{2}$ ,1), [=(1, $\omega^{2}$ , $\omega^{2}$ ,1,1,1), (=( ω ,1, $\omega^{2}$ ,1,1, ω ), x=( $\omega^{2}$ , ω ,1, ω ,1,1), !=( ω ,1, $\omega^{2}$ ,1, ω ,1), X=( $\omega^{2}$ , ω ,1,1, ω ,1), q=( $\omega^{2}$ , ω ,1,1,1, ω ), 2=(1, $\omega^{2}$ , ω , ω ,1,1), c=( ω ,1, $\omega^{2}$ , ω ,1,1), B=(1, $\omega^{2}$ , ω ,1, ω ,1), I=(1, $\omega^{2}$ , ω ,1,1, ω ), <=( ω ,1, ω ,1,1, $\omega^{2}$ ), $=( ω ,1, ω ,1, $\omega^{2}$ ,1), -=(1, $\omega^{2}$ ,1, $\omega^{2}$ ,1,1), j=( ω ,1, ω , $\omega^{2}$ ,1,1), N=(1, $\omega^{2}$ ,1, ω , ω ,1), S=(1, $\omega^{2}$ ,1, ω ,1, ω ), v=(1, $\omega^{2}$ ,1,1, $\omega^{2}$ ,1), V=(1, $\omega^{2}$ ,1,1, ω , ω ), o=(1, $\omega^{2}$ ,1,1,1, $\omega^{2}$ ), )=( ω , $\omega^{2}$ ,1,1,1, ω ), "=( $\omega^{2}$ ,1, ω , ω ,1,1), y=( ω , $\omega^{2}$ ,1,1, ω ,1), d=( $\omega^{2}$ ,1, ω ,1, ω ,1), r=( $\omega^{2}$ ,1, ω ,1,1, ω ), C=(1, ω , $\omega^{2}$ , ω ,1,1), Y=( ω , $\omega^{2}$ ,1, ω ,1,1), 3=(1, ω , $\omega^{2}$ ,1, ω ,1), 7=(1, ω , $\omega^{2}$ ,1,1, ω ), k=( $\omega^{2}$ ,1,1, ω , ω ,1), t=( $\omega^{2}$ ,1,1, ω ,1, ω ), O=(1, ω , ω , $\omega^{2}$ ,1,1), :=( $\omega^{2}$ ,1,1,1, ω , ω ), >=( $\omega^{2}$ ,1,1,1,1, $\omega^{2}$ ), &=( $\omega^{2}$ ,1,1,1, $\omega^{2}$ ,1), Q=(1, ω , ω ,1, $\omega^{2}$ ,1), n=( $\omega^{2}$ ,1,1, $\omega^{2}$ ,1,1), U=(1, ω , ω ,1,1, $\omega^{2}$ ), a=( ω , $\omega^{2}$ , ω ,1,1,1), 8=(1, ω ,1, $\omega^{2}$ , ω ,1), 4=(1, ω ,1, $\omega^{2}$ ,1, ω ), F=(1, ω ,1, ω , $\omega^{2}$ ,1), i=( $\omega^{2}$ ,1, $\omega^{2}$ ,1,1,1), L=(1, ω ,1, ω ,1, $\omega^{2}$ ), D=(1, ω ,1,1, $\omega^{2}$ , ω ), J=(1, ω ,1,1, ω , $\omega^{2}$ ), *=(1,1,1,1,1, ω ), z=(1,1,1,1, ω ,1), #=(1,1,1,1, $\omega^{2}$ , $\omega^{2}$ ), e=(1, ω ,1,1,1,1), b=(1,1,1, ω ,1,1), 5=(1,1,1, ω , ω , $\omega^{2}$ ), 9=(1,1,1, ω , $\omega^{2}$ , ω ), f=(1,1,1, $\omega^{2}$ ,1, $\omega^{2}$ ), G=(1,1,1, $\omega^{2}$ , ω , ω ), s=(1,1,1, $\omega^{2}$ , $\omega^{2}$ ,1), Z=(1,1, ω ,1,1,1), A=(1,1, ω ,1, ω , $\omega^{2}$ ), 6=(1,1, ω ,1, $\omega^{2}$ , ω ), H=(1,1, ω , ω ,1, $\omega^{2}$ ), g=( $\omega^{2}$ , $\omega^{2}$ ,1,1,1,1), M=(1,1, ω , ω , $\omega^{2}$ ,1), E=(1,1, ω , $\omega^{2}$ ,1, ω ), K=(1,1, ω , $\omega^{2}$ , ω ,1), h=( ω , ω , $\omega^{2}$ ,1,1,1), ==( ω , ω ,1,1,1, $\omega^{2}$ ), %=( ω , ω ,1,1, $\omega^{2}$ ,1), l=(1,1, $\omega^{2}$ ,1,1, $\omega^{2}$ ), R=(1,1, $\omega^{2}$ ,1, ω , ω ), u=(1,1, $\omega^{2}$ ,1, $\omega^{2}$ ,1), /=(1,1, $\omega^{2}$ , $\omega^{2}$ ,1,1),    m=( ω , ω ,1, $\omega^{2}$ ,1,1),    P=(1,1, $\omega^{2}$ , ω ,1, ω ), T=(1,1, $\omega^{2}$ , ω , ω ,1)

**31-16** 237,7HG,GTUVRP,PRNQSM,MIJKL2,235,7235,3NOKLM,WXJRSM,WYZGRV,aOQbcM,YdHce,XIfbcM,fgUHce,gTUGH,YZdGH.

**44-16** (master for **31-16**) 123456,72389A,723B5C,7DEFGH,2IJKLM,3NOKLM,PNQRSM,PTUGRV,WXJRSM,WYZGRV,aOQbcM,aYdHce,XIfbcM,fgUHce,gTUhGH,iYZdGH. 1=(0,0,1,−1,1,0), 7=(0,0,−1,1,1,0), 2=(0,1,0,0,0,1), 3=(0,1,0,0,0,−1), P=(0,1,0,0,1,0), W=(0,1,0,0,−1,0), a=(0,1,0,1,0,0), X=(0,1,0,1,1,1), D=(0,1,0,1,−1,0), N=(0,1,0,1,−1,1), I=(0,1,0,1,−1,−1), f=(0,1,0,−1,0,0), O=(0,1,0,−1,1,1), J=(0,1,0,−1,1,−1), Q=(0,−1,0,1,1,1), E=(0,1,1,0,1,0), g=(0,1,1,1,1,0), i=(0,1,1,1,−1,0), Y=(0,1,1,−1,1,0), T=(0,1,1,−1,−1,0), Z=(0,1,−1,1,1,0), U=(0,1,−1,1,−1,0), F=(0,−1,1,1,0,0), h=(0,1,−1,−1,1,0), d=(0,−1,1,1,1,0), G=(1,0,0,0,0,1), H=(1,0,0,0,0,−1), 8=(1,0,0,1,−1,0), B=(1,0,0,−1,1,0), 4=(−1,0,0,1,1,0), 9=(1,0,1,0,1,0), b=(1,0,1,0,1,−1), c=(1,0,1,0,−1,1), 5=(1,0,1,1,0,0), R=(1,0,1,1,0,−1), K=(1,0,1,1,1,0), S=(1,0,1,−1,0,1), L=(1,0,1,−1,−1,0), M=(1,0,−1,0,0,0), 6=(1,0,−1,0,1,0), e=(1,0,−1,0,1,1), C=(−1,0,1,0,1,0), A=(−1,0,1,1,0,0), V=(−1,0,1,1,0,1)

**117-116** (stripmaster of the 236-1216 supermaster) 123456,123789,1AB4CD,1AB7EF,1GHI5F,1GHJC9,1KLI8D,1KLJE6,MN8COP,MNF6QR,STE5OP,ST9DQR,UVWXYZ,UVabcd,UefgYZ,Uefabh,Uijgcd,UijWXh,UBk4XZ,UBk7ac,U3l4bd,U3l7WY,ULmIXY,ULmJbc,UHnIad,UHnJWZ,opVgYZ,opVabh,opijgh,opabqR,opYZPr,oistuv,oiwxyz,oj!"#$,oj%&’(,)*Vgcd,)*VWXh,)*efgh,)*WXqR,)*cdPr,)e-/uv,)e:;yz,)f!"<=,)f%&>?,*e@[#$,*e\]’(,*fst^_,*fwx‘{,pi@[<=,pi\]>?,pj-/^_,pj:;‘{,2|V4XZ,2|V7ac,2|3l47,2|ac}∼,2|XZ+1+2,2389u{,2356^z,2l\"+3+4,2l%[+5+6,A+7V4bd,A+7V7WY, A+7Bk47,A+7WY}∼,A+7bd+1+2,ABEFu{,ABCD^z,Ak\"+8+9,Ak%[+A+B,G+CVIXY,G+CVJbc,G+CHnIJ,G+Cbc+D+E,G+CXY+F+G,GHC9#?,GH5F<(,Gn:t+3+B,Gnw/+8+6,K+HVIad,K+HVJWZ,K+HLmIJ,K+HWZ+D+E,K+Had+F+G,KLE6#?,KL8D<(,Km:t+5+9,Kmw/+A+4,+7B@&+3+4,+7B!]+5+6,+7k89y_,+7k56‘v,|3@&+8+9,|3!]+A+B,|lEFy_,|lCD‘v,+HL-x+3+B,+HLs;+8+6,+HmC9’=,+Hm5F>$,+CH-x+5+9,+CHs;+A+4,+CnE6’=,+Cn8D>$,efab+IO,efYZQ+J,ijWX+IO,ijcdQ+J,Bkac+K+L, BkXZ+M+N,3lWY+K+L,3lbd+M+N,Lmbc+O+P,LmXY+Q+R,HnWZ+O+P,Hnad+Q+R. 1=(0,1,0,−1,0,0), M=(0,1,0,−1,1,1), S=(0,1,0,−1,1,−1), T=(0,1,0,−1,−1,1), N=(0,−1,0,1,1,1), U=(0,1,1,0,0,0), o=(0,1,1,0,1,1), )=(0,1,1,0,1,−1), *=(0,1,1,0,−1,1), p=(0,1,1,0,−1,−1), 2=(0,1,1,1,0,1), A=(0,1,1,1,0,−1), G=(0,1,1,1,1,0), K=(0,1,1,1,−1,0), +7=(0,1,1,−1,0,1), |=(0,1,1,−1,0,−1), +H=(0,1,1,−1,1,0), +C=(0,1,1,−1,−1,0), V=(0,1,−1,0,0,0), e=(0,1,−1,0,1,1), i=(0,1,−1,0,1,−1), j=(0,1,−1,0,−1,1), f=(0,−1,1,0,1,1), B=(0,1,−1,1,0,1), 3=(0,1,−1,1,0,−1), L=(0,1,−1,1,1,0), H=(0,1,−1,1,−1,0), l=(0,1,−1,−1,0,1), k=(0,−1,1,1,0,1), n=(0,1,−1,−1,1,0), m=(0,−1,1,1,1,0), I=(1,0,0,0,0,1), J=(1,0,0,0,0,−1), 4=(1,0,0,0,1,0), 7=(1,0,0,0,−1,0), g=(1,0,0,1,0,0), W=(1,0,0,1,1,1), a=(1,0,0,1,1,−1), b=(1,0,0,1,−1,1), X=(1,0,0,1,−1,−1), h=(1,0,0,−1,0,0), c=(1,0,0,−1,1,1), Y=(1,0,0,−1,1,−1), Z=(1,0,0,−1,−1,1), d=(−1,0,0,1,1,1), E=(1,0,1,0,1,1), 8=(1,0,1,0,1,−1), C=(1,0,1,0,−1,1), 5=(1,0,1,0,−1,−1), −=(1,0,1,1,0,1), s=(1,0,1,1,0,−1), @=(1,0,1,1,1,0), !=(1,0,1,1,−1,0), :=(1,0,1,−1,0,1), w=(1,0,1,−1,0,−1), \=(1,0,1,−1,1,0), %=(1,0,1,−1,−1,0), 9=(1,0,−1,0,1,1), F=(1,0,−1,0,1,−1), 6=(1,0,−1,0,−1,1), D=(−1,0,1,0,1,1), t=(1,0,−1,1,0,1), /=(1,0,−1,1,0,−1), "=(1,0,−1,1,1,0), [=(1,0,−1,1,−1,0), x=(1,0,−1,−1,0,1), ;=(−1,0,1,1,0,1), &=(1,0,−1,−1,1,0), ]=(−1,0,1,1,1,0), +A=(1,1,0,0,1,1), +5=(1,1,0,0,1,−1), +8=(1,1,0,0,−1,1), +3=(1,1,0,0,−1,−1), >=(1,1,0,1,0,1), ’=(1,1,0,1,0,−1), ‘=(1,1,0,1,1,0), y=(1,1,0,1,−1,0), <=(1,1,0,−1,0,1), #=(1,1,0,−1,0,−1), ^=(1,1,0,−1,1,0), u=(1,1,0,−1,−1,0), +Q=(1,1,1,0,0,1), +O=(1,1,1,0,0,−1), +M=(1,1,1,0,1,0), +K=(1,1,1,0,−1,0), Q=(1,1,1,1,0,0), +I=(1,1,1,−1,0,0), O=(−1,1,1,1,0,0), +F=(1,1,−1,0,0,1), +D=(1,1,−1,0,0,−1), +1=(1,1,−1,0,1,0), }=(1,1,−1,0,−1,0), P=(1,1,−1,1,0,0), +J=(1,−1,−1,1,0,0), q=(1,1,−1,−1,0,0), +L=(−1,1,1,0,1,0), +N=(1,−1,−1,0,1,0), +6=(1,−1,0,0,1,1), +B=(1,−1,0,0,1,−1), +4=(1,−1,0,0,−1,1), +9=(−1,1,0,0,1,1), (=(1,−1,0,1,0,1), ?=(1,−1,0,1,0,−1), z=(1,−1,0,1,1,0), {=(1,−1,0,1,−1,0), $=(1,−1,0,−1,0,1), ==(−1,1,0,1,0,1), v=(1,−1,0,−1,1,0), _=(−1,1,0,1,1,0), +G=(1,−1,1,0,0,1), +E=(1,−1,1,0,0,−1), +2=(1,−1,1,0,1,0), ∼=(1,−1,1,0,−1,0), r=(1,−1,1,1,0,0), +P=(−1,1,1,0,0,1), +R=(1,−1,−1,0,0,1), R=(1,−1,1,−1,0,0)

### Appendix A.5. Seven-dim MMPHs

**14-8**   12567,189A5BC,189DE7,189HJ,189HB,2D,2EC,AJ.

**31-13** 1234,189DEF,189GHIJ,189KHBL,2MNDOIP,2MNEOCL,2MNGKF,QRNSAJP,QT4U,RTV9,WXMS,WYV8AJP,XY3U.

**34-14** (master for **14-8** and **31-13**) 1234567,189A5BC,189DE7F,189GHIJ,189KHBL,2MNDOIP,2MNEOCL,2MNGK6F,QRNSAJP,QT4U567,RTV9567,WXMS567,WYV8AJP,XY3U567. 1=(0,0,0,1,0,0,0); 2=(0,0,1,0,0,0,0); 3=(1,−1,0,0,0,0,0); 4=(1,1,0,0,0,0,0); 5=(0,0,0,0,0,0,1);6=(0,0,0,0,1,1,0); 7=(0,0,0,0,1,−1,0); 8=(0,1,−1,0,0,0,0); 9=(0,1,1,0,0,0,0); A=(0,0,0,0,1,0,0);B=(1,0,0,0,0,−1,0); C=(1,0,0,0,0,1,0); D=(1,0,0,0,1,1,−1); E=(−1,0,0,0,1,1,1); F=(1,0,0,0,0,0,1); G=(1,0,0,0,1,−1,−1);H=(1,0,0,0,1,1,1);I=(1,0,0,0,−1,0,0);J=(0,0,0,0,0,1,−1);K=(1,0,0,0,−1,1,−1); L=(0,0,0,0,1,0,−1); M=(0,1,0,1,0,0,0); N=(0,1,0,−1,0,0,0); O=(1,0,0,0,1,−1,1); P=(0,0,0,0,0,1,1); Q=(−1,1,1,1,0,0,0); R=(1,1,−1,1,0,0,0); S=(1,0,1,0,0,0,0); T=(1,−1,1,1,0,0,0); U=(0,0,1,−1,0,0,0); V=(1,0,0,−1,0,0,0); W=(1,−1,−1,1,0,0,0); X=(1,1,−1,−1,0,0,0); Y=(1,1,1,1,0,0,0)

### Appendix A.6. Eight-dim MMPHs

**15-9** 17426538,8E,E2M,M3N,N4O,O5U,U7Q,Q6H,H1.

**36-9** (master for **15-9**) 17426538,8ABDF9CE,ELaR2YVM,M3WSDKZN,NCJXR4TO,OV5PSBIU,UZ9GXa7Q,QTY6PWAH,HIKFGJL1. 1=(0,0,0,0,0,0,0,1), 2=(0,0,0,0,0,0,1,0), 3=(0,0,0,0,0,1,0,0), 4=(0,0,0,0,1,0,0,0), 5=(0,0,1,1,0,0,0,0), 6=(0,0,−1,1,0,0,0,0), 7=(1,1,0,0,0,0,0,0), 8=(−1,1,0,0,0,0,0,0), 9=(0,0,0,0,0,0,1,1), A=(0,0,1,1,1,−1,0,0), B=(1,1,0,0,0,0,−1,1), C=(1,1,0,0,0,0,1,−1), D=(0,0,−1,0,1,0,0,0), E=(0,0,0,1,0,1,0,0), F=(0,0,1,−1,1,1,0,0), G=(0,0,0,1,1,0,0,0), H=(0,0,1,1,−1,1,0,0), I=(1,0,0,0,0,0,1,0), J=(0,0,−1,0,0,1,0,0), K=(−1,0,0,0,0,0,1,0), L=(0,1,0,0,0,0,0,0), M=(0,0,1,0,1,0,0,0), N=(0,0,0,1,0,0,0,0), O=(−1,1,0,0,0,0,1,1), P=(0,0,0,0,1,1,0,0), Q=(−1,1,0,0,0,0,1,−1), R=(1,0,0,0,0,0,0,1), S=(0,−1,0,0,0,0,0,1), T=(0,−1,0,0,0,0,1,0), U=(0,0,−1,1,−1,1,0,0), V=(0,0,−1,1,1,−1,0,0), W=(1,1,0,0,0,0,1,1), X=(0,0,1,0,0,1,0,0), Y=(−1,0,0,0,0,0,0,1), Z=(−1,1,0,0,0,0,−1,1), a=(0,0,−1,−1,1,1,0,0)

### Appendix A.7. Nine-dim MMPHs

**13-6**    SU,1G42U,1S,472acefhK,G72,4U.

**44-6** (**13-6** filled)    SUCDEFOQ6,1G42U8H95,1SAIMSVXbi,472acefhK,G72LWYZdg,4UBJ3NPRT. 1=(0,0,0,0,0,0,0,1,0); 2=(0,0,0,0,0,0,1,0,0); 3=(0,0,0,0,1,1,0,0,0); 4=(0,0,0,1,0,0,0,0,1); 5=(0,0,0,1,0,0,0,0,−1); 6=(0,0,0,1,0,0,−1,1,0); 7=(0,0,1,0,0,0,0,1,0); 8=(0,0,1,0,0,1,0,0,0);9=(0,0,1,0,0,−1,0,0,0); A=(0,0,1,0,−1,0,1,0,0); B=(0,0,1,0,−1,1,1,0,0); C=(0,0,−1,−1,1,1,0,1,1); D=(0,0,1,−1,−1,−1,0,1,1); H=(0,1,0,0,1,0,0,0,0); E=(0,1,0,0,1,−1,1,1,−1);F=(0,1,0,0,1,−1,−1,−1,1); G=(0,1,0,0,−1,0,0,0,0); I=(0,1,0,1,1,1,1,0,1); J=(0,1,0,−1,0,0,0,−1,1); K=(1,−1,1,1,−1,1,0,−1,−1); L=(0,1,0,−1,1,1,0,0,0); M=(0,1,0,−1,1,−1,1,0,−1);N=(0,1,−1,0,0,0,1,1,0); O=(0,1,1,1,0,1,1,0,1); P=(0,1,1,1,1,−1,1,−1,−1);Q=(0,1,1,−1,0,1,−1,0,−1); R=(0,1,−1,0,0,0,1,1,0); S=(0,−1,1,0,1,0,0,0,0); U=(1,0,0,0,0,0,0,0,0); V=(1,0,0,0,0,−1,0,0,1); W=(1,0,0,1,0,1,0,0,1); X=(1,0,0,−1,0,1,0,0,0); Y=(1,0,0,−1,0,−1,0,0,1); Z=(1,0,1,0,0,0,0,−1,−1); a=(1,1,0,0,−1,−1,0,0,0); b=(1,1,1,1,0,0,−1,0,−1);c=(1,1,1,−1,1,1,0,−1,1); d=(−1,1,1,1,1,−1,0,−1,1); e=(1,−1,−1,−1,−1,1,0,1,1);f=(1,1,−1,1,1,1,0,1,−1); g=(1,1,−1,1,1,−1,0,1,−1); h=(1,−1,0,0,1,−1,0,0,0);i=(1,−1,−1,1,0,0,1,0,−1).

**19-8**    1234567,129ABCDE,13FGH5IJE,GB6E,AJE,97E,LH4DE,FIE.

**47-16** (master for **19-8**) 123456789,12ABCDEF,13HIJ5KL,1AMCLNOPQ,1BRSETUVQ,1H4567VWX,1ICDEFYOX,1ZJ4FTUW9,1ZCDEFYP8,23abRSET,cdIeMD6f,cgaZMCLN,dghA4567,ijhBRk7f,ilbZJ4FT,jlHeSkKN. 1=(1,0,0,0,0,0,0,0,0), 2=(0,1,0,0,0,0,0,0,0), 3=(0,0,1,0,0,0,0,0,0), c=(1,1,1,1,0,0,0,0,0), d=(1,−1,1,−1,0,0,0,0,0), g=(1,−1,−1,1,0,0,0,0,0), i=(1,−1,−1,−1,0,0,0,0,0), j=(1,−1,1,1,0,0,0,0,0), l=(1,1,1,−1,0,0,0,0,0), h=(1,1,0,0,0,0,0,0,0), A=(0,0,1,1,0,0,0,0,0), B=(0,0,1,−1,0,0,0,0,0), H=(0,1,0,1,0,0,0,0,0), I=(0,1,0,−1,0,0,0,0,0), e=(1,0,−1,0,0,0,0,0,0), a=(1,0,0,−1,0,0,0,0,0), b=(1,0,0,1,0,0,0,0,0), Z=(0,1,−1,0,0,0,0,0,0), J=(0,0,0,0,1,0,0,0,0), 4=(0,0,0,0,0,1,0,0,0), 5=(0,0,0,0,0,0,1,0,0), M=(0,0,0,0,1,1,1,1,0), C=(0,0,0,0,1,−1,1,−1,0), D=(0,0,0,0,1,−1,−1,1,0), R=(0,0,0,0,1,−1,−1,−1,0), S=(0,0,0,0,1,−1,1,1,0), k=(0,0,0,0,1,1,1,−1,0), E=(0,0,0,0,1,1,0,0,0), F=(0,0,0,0,0,0,1,1,0), T=(0,0,0,0,0,0,1,−1,0), K=(0,0,0,0,0,1,0,1,0), L=(0,0,0,0,0,1,0,−1,0), N=(0,0,0,0,1,0,−1,0,0), 6=(0,0,0,0,1,0,0,−1,0), 7=(0,0,0,0,1,0,0,1,0), f=(0,0,0,0,0,1,−1,0,0), Y=(0,1,1,1,0,0,0,0,1), O=(0,1,−1,1,0,0,0,0,−1), P=(0,1,1,−1,0,0,0,0,−1), U=(0,1,1,1,0,0,0,0,−1), V=(0,1,−1,−1,0,0,0,0,−1), W=(0,1,1,−1,0,0,0,0,1), Q=(0,1,0,0,0,0,0,0,1), X=(0,0,1,0,0,0,0,0,−1), 8=(0,0,0,1,0,0,0,0,−1), :9=(0,0,0,1,0,0,0,0,1)

### Appendix A.8. Ten-dim MMPHs

**18-9**    1BC5DEFGH9,1BCKL9A,T5DEU,TPR,TbL9A,CbKP,bKG,bKFR,bKUHA.

**50-15** (master for **18-9**) 12BCUVfgik,1DEJXYceoq,1DELMVWajk,1DEMNRSblm,1DEOPRTajk,1DEOQYZajk,2GHLNXZajk,2GHPQUWblm,45EFUVcdmn,46GIJSTajk,46GIUVceoq,56ABUVdeij,78ACUVfhpq,79HIUVabop,89DFUVghln. 1=(1,0,0,0,0,0,0,0,0,0), 2=(0,1,0,0,0,0,0,0,0,0), 3=(0,0,1,0,0,0,0,0,0,0), 4=(1,1,1,1,0,0,0,0,0,0), 5=(1,−1,1,−1,0,0,0,0,0,0), 6=(1,−1,−1,1,0,0,0,0,0,0), 7=(1,−1,−1,−1,0,0,0,0,0,0), 8=(1,−1,1,1,0,0,0,0,0,0), 9=(1,1,1,−1,0,0,0,0,0,0), A=(1,1,0,0,0,0,0,0,0,0), B=(0,0,1,1,0,0,0,0,0,0), C=(0,0,1,−1,0,0,0,0,0,0), D=(0,1,0,1,0,0,0,0,0,0), E=(0,1,0,−1,0,0,0,0,0,0), F=(1,0,−1,0,0,0,0,0,0,0), G=(1,0,0,−1,0,0,0,0,0,0), H=(1,0,0,1,0,0,0,0,0,0), I=(0,1,−1,0,0,0,0,0,0,0), J=(0,0,0,0,1,0,0,0,0,0), K=(0,0,0,0,0,1,0,0,0,0), L=(0,0,1,0,1,1,1,0,0,0), M=(0,0,1,0,−1,1,−1,0,0,0), N=(0,0,1,0,−1,−1,1,0,0,0), O=(0,0,1,0,−1,−1,−1,0,0,0), P=(0,0,1,0,−1,1,1,0,0,0), Q=(0,0,1,0,1,1,−1,0,0,0), R=(0,0,1,0,1,0,0,0,0,0), S=(0,0,0,0,0,1,1,0,0,0), T=(0,0,0,0,0,1,−1,0,0,0), U=(0,0,0,0,1,0,1,0,0,0), V=(0,0,0,0,1,0,−1,0,0,0), W=(0,0,1,0,0,−1,0,0,0,0), X=(0,0,1,0,0,0,−1,0,0,0), Y=(0,0,1,0,0,0,1,0,0,0), Z=(0,0,0,0,1,−1,0,0,0,0), a=(0,0,0,0,0,0,0,1,0,0), b=(0,0,0,0,0,0,0,0,1,0), c=(0,0,0,0,0,1,0,1,1,1), d=(0,0,0,0,0,1,0,−1,1,−1), e=(0,0,0,0,0,1,0,−1,−1,1), f=(0,0,0,0,0,1,0,−1,−1,−1), g=(0,0,0,0,0,1,0,−1,1,1), h=(0,0,0,0,0,1,0,1,1,−1), i=(0,0,0,0,0,1,0,1,0,0), j=(0,0,0,0,0,0,0,0,1,1), k=(0,0,0,0,0,0,0,0,1,−1), l=(0,0,0,0,0,0,0,1,0,1), m=(0,0,0,0,0,0,0,1,0,−1), n=(0,0,0,0,0,1,0,0,−1,0), o=(0,0,0,0,0,1,0,0,0,−1), p=(0,0,0,0,0,1,0,0,0,1),      q=(0,0,0,0,0,0,0,1,−1,0)

### Appendix A.9. Eleven-dim MMPHs

**19-8**   123456789AB,1234567CDF,1GHKLMDA,27KL9,567MC,567B,H8F,G8F.

**50-14** (master for **19-8**) 123456789AB,1234567CDEF,1GHIJKLMDAN,1GHIJKLOPQR,27STUVKL8FW,27STUVKL9QX,347YZabMDAN,567cdefMCXR,567cdefgOEW,567cdefgPBN,cdhijkJV8FW,eYlmnIUk9QX,fZoHTjmn8FW,abGShilo8FW. 1=(0,0,1,1,1,1,0,0,0,0,0), 2=(0,0,1,−1,1,−1,0,0,0,0,0), 3=(0,0,0,1,0,−1,0,0,0,0,0), 4=(0,0,1,0,−1,0,0,0,0,0,0), 5=(0,1,0,0,0,0,0,0,0,0,0), 6=(1,0,0,0,0,0,0,0,0,0,0), 7=(0,0,0,0,0,0,1,0,0,0,0), 8=(0,0,0,1,0,0,0,0,0,0,0), 9=(0,0,1,0,0,0,0,0,0,0,0), A=(0,0,0,0,0,1,0,0,0,0,0), B=(0,0,0,0,1,0,0,0,0,0,0), C=(1,−1,1,0,1,0,0,0,0,0,0), D=(1,1,0,1,0,1,0,0,0,0,0), E=(1,1,0,−1,0,−1,0,0,0,0,0), F=(−1,1,1,0,1,0,0,0,0,0,0), G=(0,1,−1,1,0,0,1,0,0,0,0), H=(1,0,1,1,0,0,0,−1,0,0,0), I=(1,0,0,0,1,1,0,1,0,0,0), J=(0,1,0,0,−1,1,−1,0,0,0,0), K=(0,0,1,0,−1,0,1,1,0,0,0), L=(0,0,0,1,0,−1,−1,1,0,0,0), M=(1,0,1,0,0,−1,1,0,0,0,0), N=(0,−1,1,0,0,1,0,1,0,0,0), O=(−1,1,0,0,0,0,1,1,0,0,0), P=(1,0,−1,−1,0,0,0,1,0,0,0), Q=(0,1,1,−1,0,0,−1,0,0,0,0), R=(1,0,0,1,−1,0,−1,0,0,0,0), S=(0,1,0,1,1,0,0,1,0,0,0), T=(1,1,0,0,0,0,1,−1,0,0,0), U=(0,1,0,0,1,−1,−1,0,0,0,0), V=(1,0,0,0,−1,−1,0,1,0,0,0), W=(1,1,0,−1,0,1,0,0,0,0,0), X=(1,−1,−1,0,1,0,0,0,0,0,0), Y=(0,0,0,0,0,0,0,0,1,0,0), Z=(0,0,0,0,0,0,0,0,0,1,0), a=(0,0,0,0,0,0,0,1,1,1,1), b=(0,0,0,0,0,0,0,1,−1,1,−1), c=(0,0,0,0,0,0,0,1,−1,−1,1), d=(0,0,0,0,0,0,0,1,−1,−1,−1), e=(0,0,0,0,0,0,0,1,−1,1,1), f=(0,0,0,0,0,0,0,1,1,1,−1), g=(0,0,0,0,0,0,0,1,1,0,0), h=(0,0,0,0,0,0,0,0,0,1,1), i=(0,0,0,0,0,0,0,0,0,1,−1), j=(0,0,0,0,0,0,0,0,1,0,1), k=(0,0,0,0,0,0,0,0,1,0,−1), l=(0,0,0,0,0,0,0,1,0,−1,0), m=(0,0,0,0,0,0,0,1,0,0,−1),   n=(0,0,0,0,0,0,0,1,0,0,1),   o=(0,0,0,0,0,0,0,0,1,−1,0)

### Appendix A.10. Twelve-dim MMPHs

**19-9**   123456789ABC,17DJBL,28PQA,PJ,3478ST,5678Q,SC,T9,DL

**52-9** (master for **19-9**) 123456789ABC,17DEFGHIJBKL,28MNOPHIQAKR,3478STUVWXYR,5678ZabcdWQe,ZafghiGPjJke,bSlmnFOijdoC,cTpENhmn9qYo,UVDMfglpkXqL. 1=(0,0,1,1,1,1,0,0,0,0,0,0), 2=(0,0,1,−1,1,−1,0,0,0,0,0,0), 3=(0,0,0,1,0,−1,0,0,0,0,0,0), 4=(0,0,1,0,−1,0,0,0,0,0,0,0), 5=(0,1,0,0,0,0,0,0,0,0,0,0), 6=(1,0,0,0,0,0,0,0,0,0,0,0), 7=(0,0,0,0,0,0,0,1,0,0,0,0), 8=(0,0,0,0,0,0,1,0,0,0,0,0), Z=(0,0,0,1,0,0,0,0,0,0,0,0), a=(0,0,1,0,0,0,0,0,0,0,0,0), b=(0,0,0,0,0,1,0,0,0,0,0,0), c=(0,0,0,0,1,0,0,0,0,0,0,0), S=(1,−1,1,0,1,0,0,0,0,0,0,0), T=(1,1,0,1,0,1,0,0,0,0,0,0), U=(1,1,0,−1,0,−1,0,0,0,0,0,0), V=(−1,1,1,0,1,0,0,0,0,0,0,0), D=(0,1,−1,1,0,0,1,0,0,0,0,0), M=(1,0,1,1,0,0,0,−1,0,0,0,0), f=(1,0,0,0,1,1,0,1,0,0,0,0), g=(0,1,0,0,−1,1,−1,0,0,0,0,0), l=(0,0,1,0,−1,0,1,1,0,0,0,0), p=(0,0,0,1,0,−1,−1,1,0,0,0,0), E=(1,0,1,0,0,−1,1,0,0,0,0,0), N=(0,−1,1,0,0,1,0,1,0,0,0,0), h=(−1,1,0,0,0,0,1,1,0,0,0,0), m=(1,0,−1,−1,0,0,0,1,0,0,0,0), n=(0,1,1,−1,0,0,−1,0,0,0,0,0), F=(1,0,0,1,−1,0,−1,0,0,0,0,0), O=(0,1,0,1,1,0,0,1,0,0,0,0), i=(1,1,0,0,0,0,1,−1,0,0,0,0), G=(0,1,0,0,1,−1,−1,0,0,0,0,0), P=(1,0,0,0,−1,−1,0,1,0,0,0,0), H=(1,1,0,−1,0,1,0,0,0,0,0,0), I=(1,−1,−1,0,1,0,0,0,0,0,0,0), j=(0,0,0,0,0,0,0,0,1,0,0,0), d=(0,0,0,0,0,0,0,0,0,1,0,0), J=(0,0,0,0,0,0,0,0,0,1,1,0), k=(0,0,0,0,0,0,0,0,0,1,−1,0), W=(0,0,0,0,0,0,0,0,1,0,1,0), Q=(0,0,0,0,0,0,0,0,1,0,−1,0), 9=(0,0,0,0,0,0,0,0,1,−1,0,0), e=(0,0,0,0,0,0,0,0,0,0,0,1), A=(0,0,0,0,0,0,0,0,1,1,1,1), B=(0,0,0,0,0,0,0,0,1,1,−1,−1), K=(0,0,0,0,0,0,0,0,1,−1,1,−1), X=(0,0,0,0,0,0,0,0,1,−1,−1,−1), q=(0,0,0,0,0,0,0,0,1,1,1,−1), Y=(0,0,0,0,0,0,0,0,1,1,−1,1), L=(0,0,0,0,0,0,0,0,1,0,0,1), o=(0,0,0,0,0,0,0,0,0,0,1,1), C=(0,0,0,0,0,0,0,0,0,0,1,−1), R=(0,0,0,0,0,0,0,0,0,1,0,−1)

### Appendix A.11. Thirteen-dim MMPHs

**19-8**    123456789ABCD,123456789EFG,12345678ILM,289EBM,34789C,56789LG,5678ABM,9FD.

**63-16** (master for **19-8**) 123456789ABCD,123456789EFGH,12345678IJKLM,17NOPQRSTUVWM,28XYZaRS9EbBM,3478cdef9bghH,3478cdef9WhiC,3478cdefIjVkM, 5678lmno9LpGq, 5678lmnoATrBM,lmstuvQa9WpFD,lmstuvQaEKwxM,ncyz!PZv9AkLM,od"OYuz!9kgiq,od"OYuz!bUj xM,efNXsty"rJwWM. 1=(0,0,1,1,1,1,0,0,0,0,0,0,0), 2=(0,0,1,−1,1,−1,0,0,0,0,0,0,0), 3=(0,0,0,1,0,−1,0,0,0,0,0,0,0), 4=(0,0,1,0,−1,0,0,0,0,0,0,0,0), 5=(0,1,0,0,0,0,0,0,0,0,0,0,0), 6=(1,0,0,0,0,0,0,0,0,0,0,0,0), 7=(0,0,0,0,0,0,0,1,0,0,0,0,0), 8=(0,0,0,0,0,0,1,0,0,0,0,0,0), l=(0,0,0,1,0,0,0,0,0,0,0,0,0), m=(0,0,1,0,0,0,0,0,0,0,0,0,0), n=(0,0,0,0,0,1,0,0,0,0,0,0,0), o=(0,0,0,0,1,0,0,0,0,0,0,0,0), c=(1,−1,1,0,1,0,0,0,0,0,0,0,0), d=(1,1,0,1,0,1,0,0,0,0,0,0,0), e=(1,1,0,−1,0,−1,0,0,0,0,0,0,0), f=(−1,1,1,0,1,0,0,0,0,0,0,0,0), N=(0,1,−1,1,0,0,1,0,0,0,0,0,0), X=(1,0,1,1,0,0,0,−1,0,0,0,0,0), s=(1,0,0,0,1,1,0,1,0,0,0,0,0), t=(0,1,0,0,−1,1,−1,0,0,0,0,0,0), y=(0,0,1,0,−1,0,1,1,0,0,0,0,0), "=(0,0,0,1,0,−1,−1,1,0,0,0,0,0), O=(1,0,1,0,0,−1,1,0,0,0,0,0,0), Y=(0,−1,1,0,0,1,0,1,0,0,0,0,0), u=(−1,1,0,0,0,0,1,1,0,0,0,0,0), z=(1,0,−1,−1,0,0,0,1,0,0,0,0,0), !=(0,1,1,−1,0,0,−1,0,0,0,0,0,0), P=(1,0,0,1,−1,0,−1,0,0,0,0,0,0), Z=(0,1,0,1,1,0,0,1,0,0,0,0,0), v=(1,1,0,0,0,0,1,−1,0,0,0,0,0), Q=(0,1,0,0,1,−1,−1,0,0,0,0,0,0), a=(1,0,0,0,−1,−1,0,1,0,0,0,0,0), R=(1,1,0,−1,0,1,0,0,0,0,0,0,0), S=(1,−1,−1,0,1,0,0,0,0,0,0,0,0), 9=(0,0,0,0,0,0,0,0,1,0,0,0,0), A=(0,0,0,0,0,0,0,0,0,1,0,0,0), E=(0,0,0,0,0,0,0,0,0,1,1,0,0), b=(0,0,0,0,0,0,0,0,0,1,−1,0,0), T=(0,0,0,0,0,0,0,0,1,0,1,0,0), r=(0,0,0,0,0,0,0,0,1,0,−1,0,0), I=(0,0,0,0,0,0,0,0,1,−1,0,0,0), B=(0,0,0,0,0,0,0,0,0,0,0,1,0), J=(0,0,0,0,0,0,0,0,1,1,1,1,0), K=(0,0,0,0,0,0,0,0,1,1,−1,−1,0), w=(0,0,0,0,0,0,0,0,1,−1,1,−1,0), U=(0,0,0,0,0,0,0,0,1,−1,−1,−1,0), j=(0,0,0,0,0,0,0,0,1,1,1,−1,0), V=(0,0,0,0,0,0,0,0,1,1,−1,1,0), x=(0,0,0,0,0,0,0,0,1,0,0,1,0), k=(0,0,0,0,0,0,0,0,0,0,1,1,0), L=(0,0,0,0,0,0,0,0,0,0,1,−1,0), W=(0,0,0,0,0,0,0,0,0,1,0,−1,0), M=(0,0,0,0,0,0,0,0,0,0,0,0,1), p=(0,0,0,0,0,0,0,0,0,1,1,1,1), F=(0,0,0,0,0,0,0,0,0,1,−1,1,−1), G=(0,0,0,0,0,0,0,0,0,1,−1,−1,1), g=(0,0,0,0,0,0,0,0,0,1,1,−1,1), h=(0,0,0,0,0,0,0,0,0,1,1,1,−1), i=(0,0,0,0,0,0,0,0,0,1,−1,1,1), H=(0,0,0,0,0,0,0,0,0,0,0,1,1), C=(0,0,0,0,0,0,0,0,0,0,1,0,1), D=(0,0,0,0,0,0,0,0,0,0,1,0,−1), q=(0,0,0,0,0,0,0,0,0,1,0,0,−1)

### Appendix A.12. Fourteen-dim MMPHs

**19-9**   123456789ABCDE,12345679ABFGD,1OPF,27a,347E,3479ABP,567CG,9a,O89AB.

**66-15** (master for **19-9**) 123456789ABCDE,12345679ABFGDH,1IJKLMNOPFQRST,27UVWXMN YZTabc,347defghijkElm,347defg9ABPHnm,347defgFijkGDH,567opqrOBPFsZt,567opqr9ABaunl,567opqrijbkCGu,opvwxyLX9iQYab,qdz!"KWyAt#Sab,re$JVx!"s%abck,fgIUvwz$Oh89AB,fgIUvwz$#R%jab. 1=(0,0,1,1,1,1,0,0,0,0,0,0,0,0), 2=(0,0,1,−1,1,−1,0,0,0,0,0,0,0,0), 3=(0,0,0,1,0,−1,0,0,0,0,0,0,0,0), 4=(0,0,1,0,−1,0,0,0,0,0,0,0,0,0), 5=(0,1,0,0,0,0,0,0,0,0,0,0,0,0), 6=(1,0,0,0,0,0,0,0,0,0,0,0,0,0), 7=(0,0,0,0,0,0,1,0,0,0,0,0,0,0), o=(0,0,0,1,0,0,0,0,0,0,0,0,0,0), p=(0,0,1,0,0,0,0,0,0,0,0,0,0,0), q=(0,0,0,0,0,1,0,0,0,0,0,0,0,0), r=(0,0,0,0,1,0,0,0,0,0,0,0,0,0), d=(1,−1,1,0,1,0,0,0,0,0,0,0,0,0), e=(1,1,0,1,0,1,0,0,0,0,0,0,0,0), f=(1,1,0,−1,0,−1,0,0,0,0,0,0,0,0), g=(−1,1,1,0,1,0,0,0,0,0,0,0,0,0), I=(0,1,−1,1,0,0,1,0,0,0,0,0,0,0), U=(1,0,1,1,0,0,0,−1,0,0,0,0,0,0), v=(1,0,0,0,1,1,0,1,0,0,0,0,0,0), w=(0,1,0,0,−1,1,−1,0,0,0,0,0,0,0), z=(0,0,1,0,−1,0,1,1,0,0,0,0,0,0), $=(0,0,0,1,0,−1,−1,1,0,0,0,0,0,0), J=(1,0,1,0,0,−1,1,0,0,0,0,0,0,0), V=(0,−1,1,0,0,1,0,1,0,0,0,0,0,0), x=(−1,1,0,0,0,0,1,1,0,0,0,0,0,0), !=(1,0,−1,−1,0,0,0,1,0,0,0,0,0,0), "=(0,1,1,−1,0,0,−1,0,0,0,0,0,0,0), K=(1,0,0,1,−1,0,−1,0,0,0,0,0,0,0), W=(0,1,0,1,1,0,0,1,0,0,0,0,0,0), y=(1,1,0,0,0,0,1,−1,0,0,0,0,0,0), L=(0,1,0,0,1,−1,−1,0,0,0,0,0,0,0), X=(1,0,0,0,−1,−1,0,1,0,0,0,0,0,0), M=(1,1,0,−1,0,1,0,0,0,0,0,0,0,0), N=(1,−1,−1,0,1,0,0,0,0,0,0,0,0,0), O=(0,0,0,0,0,0,0,0,0,0,1,0,0,0), h=(0,0,0,0,0,0,0,0,1,−1,0,0,0,0), 8=(0,0,0,0,0,0,0,0,1,1,0,0,0,0), 9=(0,0,0,0,0,0,0,0,0,0,0,0,0,1), A=(0,0,0,0,0,0,0,0,0,0,0,1,1,0), B=(0,0,0,0,0,0,0,0,0,0,0,1,−1,0), P=(0,0,0,0,0,0,0,1,0,−1,0,0,0,0), F=(0,0,0,0,0,0,0,1,0,1,0,0,0,0), i=(0,0,0,0,0,0,0,0,0,0,0,1,0,0), Q=(0,0,0,0,0,0,0,0,1,0,0,0,−1,0), Y=(0,0,0,0,0,0,0,0,1,0,0,0,1,0), s=(0,0,0,0,0,0,0,0,1,0,0,1,1,−1), Z=(0,0,0,0,0,0,0,0,1,0,0,−1,−1,−1), t=(0,0,0,0,0,0,0,0,1,0,0,0,0,1), #=(0,0,0,0,0,0,0,0,1,0,0,1,−1,−1), R=(0,0,0,0,0,0,0,0,1,0,0,1,1,1), %=(0,0,0,0,0,0,0,0,1,0,0,−1,0,0), j=(0,0,0,0,0,0,0,0,0,0,0,0,1,−1), S=(0,0,0,0,0,0,0,0,1,0,0,−1,1,−1), T=(0,0,0,0,0,0,0,0,0,0,0,1,0,−1), a=(0,0,0,0,0,0,0,0,0,1,1,0,0,0), b=(0,0,0,0,0,0,0,0,0,1,−1,0,0,0), c=(0,0,0,0,0,0,0,0,1,0,0,1,−1,1), k=(0,0,0,0,0,0,0,0,0,0,0,0,1,1), C=(0,0,0,0,0,0,0,1,−1,1,1,0,0,0), G=(0,0,0,0,0,0,0,1,−1,−1,−1,0,0,0), u=(0,0,0,0,0,0,0,1,1,0,0,0,0,0), D=(0,0,0,0,0,0,0,1,1,−1,1,0,0,0), E=(0,0,0,0,0,0,0,1,0,0,−1,0,0,0), H=(0,0,0,0,0,0,0,0,1,0,−1,0,0,0), n=(0,0,0,0,0,0,0,1,−1,1,−1,0,0,0), l=(0,0,0,0,0,0,0,1,−1,−1,1,0,0,0), m=(0,0,0,0,0,0,0,1,1,1,1,0,0,0)

### Appendix A.13. Fifteen-dim MMPHs

**25-8** 123456789ABCDEF,1234567GHIJKLD,1RS89ABCDEF,27TRSX9GH,347CL,347BK,347AIJ,T8X9.

**66-14** (master for **25-8**) 123456789ABCDEF,1234567GHIJKLDM,1NOPQRS89ABCDEF,27TUVWRSX9YGHZa,347bcdeZfghiCjL,347bcdeaklBmKhi,347bcdenoApIJgl,567qrstuYpmjMEF, 567qrstvw9Yfkno,qrxyz!QW8uvwX9Y,qrxyz!QWX9YGHZa,sb"#$PV!8uvwX9Y,tc%OUz#$8uvwX9Y,deNTxy"%8uvwX9Y. 1=(0,0,1,1,1,1.0,0,0,0,0,0,0,0,0), 2=(0,0,1,−1,1,−1.0,0,0,0,0,0,0,0,0), 3=(0,0,0,1,0,−1.0,0,0,0,0,0,0,0,0), 4=(0,0,1,0,−1,0.0,0,0,0,0,0,0,0,0), 5=(0,1,0,0,0,0.0,0,0,0,0,0,0,0,0), 6=(1,0,0,0,0,0.0,0,0,0,0,0,0,0,0), 7=(0,0,0,0,0,0.1,0,0,0,0,0,0,0,0), q=(0,0,0,1,0,0.0,0,0,0,0,0,0,0,0), r=(0,0,1,0,0,0.0,0,0,0,0,0,0,0,0), s=(0,0,0,0,0,1.0,0,0,0,0,0,0,0,0), t=(0,0,0,0,1,0.0,0,0,0,0,0,0,0,0), b=(1,−1,1,0,1,0.0,0,0,0,0,0,0,0,0), c=(1,1,0,1,0,1.0,0,0,0,0,0,0,0,0), d=(1,1,0,−1,0,−1,0,0,0,0,0,0,0,0,0), e=(−1,1,1,0,1,0.0,0,0,0,0,0,0,0,0), N=(0,1,−1,1,0,0.1,0,0,0,0,0,0,0,0), T=(1,0,1,1,0,0,0,−1,0,0,0,0,0,0,0), x=(1,0,0,0,1,1.0,1,0,0,0,0,0,0,0), y=(0,1,0,0,−1,1.−1,0,0,0,0,0,0,0,0), "=(0,0,1,0,−1,0.1,1,0,0,0,0,0,0,0), %=(0,0,0,1,0,−1.−1,1,0,0,0,0,0,0,0), O=(1,0,1,0,0,−1.1,0,0,0,0,0,0,0,0), U=(0,−1,1,0,0,1.0,1,0,0,0,0,0,0,0), z=(−1,1,0,0,0,0.1,1,0,0,0,0,0,0,0), #=(1,0,−1,−1,0,0.0,1,0,0,0,0,0,0,0), $=(0,1,1,−1,0,0.−1,0,0,0,0,0,0,0,0), P=(1,0,0,1,−1,0.−1,0,0,0,0,0,0,0,0), V=(0,1,0,1,1,0.0,1,0,0,0,0,0,0,0), !=(1,1,0,0,0,0.1,−1,0,0,0,0,0,0,0), Q=(0,1,0,0,1,−1.−1,0,0,0,0,0,0,0,0), W=(1,0,0,0,−1,−1.0,1,0,0,0,0,0,0,0), R=(1,1,0,−1,0,1.0,0,0,0,0,0,0,0,0), S=(1,−1,−1,0,1,0.0,0,0,0,0,0,0,0,0), 8=(0,0,0,0,0,0.0,0,0,1,1,1,1,0,0), u=(0,0,0,0,0,0.0,0,0,1,−1,1,−1,0,0), v=(0,0,0,0,0,0.0,0,0,0,1,0,−1,0,0), w=(0,0,0,0,0,0.0,0,0,1,0,−1,0,0,0), X=(0,0,0,0,0,0.0,0,1,0,0,0,0,0,0), 9=(0,0,0,0,0,0.0,0,0,0,0,0,0,0,1), Y=(0,0,0,0,0,0.0,0,0,0,0,0,0,1,0), G=(0,0,0,0,0,0.0,0,0,0,1,0,0,0,0), H=(0,0,0,0,0,0.0,0,0,1,0,0,0,0,0), Z=(0,0,0,0,0,0.0,0,0,0,0,0,1,0,0), a=(0,0,0,0,0,0.0,0,0,0,0,1,0,0,0), f=(0,0,0,0,0,0.0,1,−1,1,0,1,0,0,0), k=(0,0,0,0,0,0.0,1,1,0,1,0,1,0,0), n=(0,0,0,0,0,0.0,1,1,0,−1,0,−1,0,0), o=(0,0,0,0,0,0.0,1,−1,−1,0,−1,0,0,0), A=(0,0,0,0,0,0.0,0,1,−1,1,0,0,1,0), p=(0,0,0,0,0,0.0,1,0,1,1,0,0,0,−1), I=(0,0,0,0,0,0.0,1,0,0,0,1,1,0,1), J=(0,0,0,0,0,0.0,0,1,0,0,−1,1,−1,0), g=(0,0,0,0,0,0.0,0,0,1,0,−1,0,1,1), l=(0,0,0,0,0,0.0,0,0,0,1,0,−1,−1,1), B=(0,0,0,0,0,0.0,1,0,1,0,0,−1,1,0), m=(0,0,0,0,0,0.0,0,1,−1,0,0,−1,0,−1), K=(0,0,0,0,0,0.0,1,−1,0,0,0,0,−1,−1), h=(0,0,0,0,0,0.0,1,0,−1,−1,0,0,0,1), i=(0,0,0,0,0,0.0,0,1,1,−1,0,0,−1,0), C=(0,0,0,0,0,0.0,1,0,0,1,−1,0,−1,0), j=(0,0,0,0,0,0.0,0,1,0,1,1,0,0,1), L=(0,0,0,0,0,0.0,1,1,0,0,0,0,1,−1), D=(0,0,0,0,0,0.0,0,1,0,0,1,−1,−1,0), M=(0,0,0,0,0,0.0,1,0,0,0,−1,−1,0,1), E=(0,0,0,0,0,0.0,1,1,0,−1,0,1,0,0), F=(0,0,0,0,0,0.0,1,−1,−1,0,1,0,0,0)

### Appendix A.14. Sixteen-dim MMPHs

**22-9**    123456789ABCDEFG,17HID,28UdG,3478efE,5678,Bd,e9,fICF,HUA.

**70-9** (master for **22-9**) 123456789ABCDEFG,17HIJKLMNOPQRDST,28UVWXLMYZabScdG,3478efghijPbkElm,5678nopqrZsQtukT,novwxyKXzasBu!dm,pe"#$JWy9iY%tR&’,qf(IVx#$zOjC)lF&,ghHUvw"(ArN%)c!’. 1=(0,0,1,1,1,1,0,0,0,0,0,0,0,0,0,0), 2=(0,0,1,−1,1,−1,0,0,0,0,0,0,0,0,0,0), 3=(0,0,0,1,0,−1,0,0,0,0,0,0,0,0,0,0), 4=(0,0,1,0,−1,0,0,0,0,0,0,0,0,0,0,0), 5=(0,1,0,0,0,0,0,0,0,0,0,0,0,0,0,0), 6=(1,0,0,0,0,0,0,0,0,0,0,0,0,0,0,0), 7=(0,0,0,0,0,0,0,1,0,0,0,0,0,0,0,0), 8=(0,0,0,0,0,0,1,0,0,0,0,0,0,0,0,0), n=(0,0,0,1,0,0,0,0,0,0,0,0,0,0,0,0), o=(0,0,1,0,0,0,0,0,0,0,0,0,0,0,0,0), p=(0,0,0,0,0,1,0,0,0,0,0,0,0,0,0,0), q=(0,0,0,0,1,0,0,0,0,0,0,0,0,0,0,0), e=(1,−1,1,0,1,0,0,0,0,0,0,0,0,0,0,0), f=(1,1,0,1,0,1,0,0,0,0,0,0,0,0,0,0), g=(1,1,0,−1,0,−1,0,0,0,0,0,0,0,0,0,0), h=(−1,1,1,0,1,0,0,0,0,0,0,0,0,0,0,0), H=(0,1,−1,1,0,0,1,0,0,0,0,0,0,0,0,0), U=(1,0,1,1,0,0,0,−1,0,0,0,0,0,0,0,0), v=(1,0,0,0,1,1,0,1,0,0,0,0,0,0,0,0), w=(0,1,0,0,−1,1,−1,0,0,0,0,0,0,0,0,0), "=(0,0,1,0,−1,0,1,1,0,0,0,0,0,0,0,0), (=(0,0,0,1,0,−1,−1,1,0,0,0,0,0,0,0,0), I=(1,0,1,0,0,−1,1,0,0,0,0,0,0,0,0,0), V=(0,−1,1,0,0,1,0,1,0,0,0,0,0,0,0,0), x=(−1,1,0,0,0,0,1,1,0,0,0,0,0,0,0,0), #=(1,0,−1,−1,0,0,0,1,0,0,0,0,0,0,0,0), $=(0,1,1,−1,0,0,−1,0,0,0,0,0,0,0,0,0), J=(1,0,0,1,−1,0,−1,0,0,0,0,0,0,0,0,0), W=(0,1,0,1,1,0,0,1,0,0,0,0,0,0,0,0), y=(1,1,0,0,0,0,1,−1,0,0,0,0,0,0,0,0), K=(0,1,0,0,1,−1,−1,0,0,0,0,0,0,0,0,0), X=(1,0,0,0,−1,−1,0,1,0,0,0,0,0,0,0,0), L=(1,1,0,−1,0,1,0,0,0,0,0,0,0,0,0,0), M=(1,−1,−1,0,1,0,0,0,0,0,0,0,0,0,0,0), 9=(0,0,0,0,0,0,0,0,1,0,0,0,0,0,0,0), A=(0,0,0,0,0,0,0,0,0,1,0,0,0,0,0,0), i=(0,0,0,0,0,0,0,0,0,1,1,0,0,0,0,0), Y=(0,0,0,0,0,0,0,0,0,1,−1,0,0,0,0,0), r=(0,0,0,0,0,0,0,0,1,0,1,0,0,0,0,0), N=(0,0,0,0,0,0,0,0,1,0,−1,0,0,0,0,0), z=(0,0,0,0,0,0,0,0,1,−1,0,0,0,0,0,0), %=(0,0,0,0,0,0,0,0,0,0,0,1,0,0,0,0), O=(0,0,0,0,0,0,0,0,1,1,1,1,0,0,0,0), j=(0,0,0,0,0,0,0,0,1,1,−1,−1,0,0,0,0), P=(0,0,0,0,0,0,0,0,1,−1,1,−1,0,0,0,0), Z=(0,0,0,0,0,0,0,0,1,−1,−1,−1,0,0,0,0), a=(0,0,0,0,0,0,0,0,1,1,1,−1,0,0,0,0), s=(0,0,0,0,0,0,0,0,1,1,−1,1,0,0,0,0), b=(0,0,0,0,0,0,0,0,1,0,0,1,0,0,0,0), B=(0,0,0,0,0,0,0,0,0,0,1,1,0,0,0,0), C=(0,0,0,0,0,0,0,0,0,0,1,−1,0,0,0,0), Q=(0,0,0,0,0,0,0,0,0,1,0,−1,0,0,0,0), t=(0,0,0,0,0,0,0,0,0,0,0,0,1,0,0,0), R=(0,0,0,0,0,0,0,0,0,0,0,0,0,1,0,0), u=(0,0,0,0,0,0,0,0,0,0,0,0,0,1,1,0), k=(0,0,0,0,0,0,0,0,0,0,0,0,0,1,−1,0), D=(0,0,0,0,0,0,0,0,0,0,0,0,1,0,1,0), S=(0,0,0,0,0,0,0,0,0,0,0,0,1,0,−1,0), )=(0,0,0,0,0,0,0,0,0,0,0,0,1,−1,0,0), T=(0,0,0,0,0,0,0,0,0,0,0,0,0,0,0,1), c=(0,0,0,0,0,0,0,0,0,0,0,0,1,1,1,1), !=(0,0,0,0,0,0,0,0,0,0,0,0,1,1,−1,−1), d=(0,0,0,0,0,0,0,0,0,0,0,0,1,−1,1,−1), E=(0,0,0,0,0,0,0,0,0,0,0,0,1,−1,−1,−1), l=(0,0,0,0,0,0,0,0,0,0,0,0,1,1,1,−1), F=(0,0,0,0,0,0,0,0,0,0,0,0,1,1,−1,1), m=(0,0,0,0,0,0,0,0,0,0,0,0,1,0,0,1), &=(0,0,0,0,0,0,0,0,0,0,0,0,0,0,1,1), ’=(0,0,0,0,0,0,0,0,0,0,0,0,0,0,1,−1), G=(0,0,0,0,0,0,0,0,0,0,0,0,0,1,0,−1)
