# Correction: Pavičić, M. Non-Kochen–Specker Contextuality. *Entropy* 2023, *25*, 1117

**DOI:** 10.3390/e26020100

**Published:** 2024-01-24

**Authors:** Mladen Pavičić

**Affiliations:** 1 Center of Excellence for Advanced Materials and Sensors, Research Unit Photonics and Quantum Optics, Institute Ruđer Bošković, 10000 Zagreb, Croatia; mpavicic@irb.hr; 2 Institute of Physics, 10000 Zagreb, Croatia

In the original publication [1], there were mistakes in Figure 3 and its caption. In the figure, the orange arrow between (h) and (i) and the green arrow between (h) and (j) as well as the text “isomorphic” are wrong. The corrected Figure 3, without these arrows and with a black arrow added to (i), appears below. The remainder of the figure stays the same as before.

Also, in its caption, “(h,i)” originally read as “(h,i) isomorphic eight-dim KS MMPHs with the smallest number of hyperedges (9) used to generate the 15-9 non-KS NBMMPH in (j).” The corrected (h,i) caption appears below.

**Figure 3:**
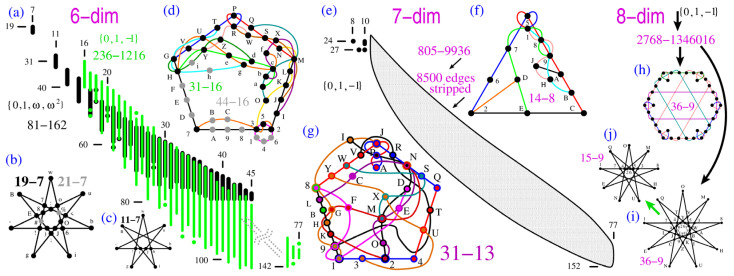
(**a**) Distributions of 6-dim critical non-KS NBMMPHs obtained from two different submasters—see text; (**b**) the smallest critical non-KS NBMMPH obtained from the former class by **M3**; it has a parity proof; (**c**) an even smaller critical non-KS NBMMPH obtained from it by hand; it has a parity proof; (**d**) the smallest critical non-KS NBMMPH obtained from the latter class by **M1**; (**e**) distributions of 7-dim critical non-KS NBMMPHs—see text; (**f**) 14-8 non-KS NBMMPH, one of the smallest non-KS NBMMPHs obtained via **M3** from the smallest KS NBMMPH 34-14; (**g**) 31-13 also obtained from the 34-14 (no m = 1 vertices essential for criticality); (**h**,**i**) two 8-dim KS MMPHs with the smallest number of hyperedges (9); (**i**) serves us in generating the 15-9 non-KS NBMMPH in (**j**); (**h**–**j**) MMPHs have parity proofs; strings and coordinatizations are given in Appendix A.

The author states that the scientific conclusions are unaffected. This correction was approved by the Academic Editor. The original publication has also been updated.
